# New challenges for the Human Oceans Past agenda

**DOI:** 10.12688/openreseurope.15095.1

**Published:** 2022-09-29

**Authors:** Poul Holm, James Barrett, Cristina Brito, Francis Ludlow

**Affiliations:** 1 Centre for Environmental Humanities, Trinity College Dublin, Dublin, D02 PN40, Ireland; 2 Department of Archaeology and Cultural History, Norwegian University of Science and Technology, Trondheim, 7491, Norway; 3 CHAM Centro de Humanidades, Universidade NOVA de Lisboa, Lisboa, 1070-312, Portugal

**Keywords:** Environmental history, archaeology, marine resources, human societies, ecological globalisation

## Abstract

We contend that the harvest of marine resources played a critical, but as yet underappreciated and poorly understood, role in global history. In a review of the field of marine environmental history and archaeology we conclude that while much progress has been made, especially in the last two decades, fundamental questions remain unanswered. In order to make full use of the rapid growth of Big Data and ongoing methodological breakthroughs there is a need for collaborative and comparative research. Such joint efforts on a global scale must be guided by a focus on common, simple yet challenging, questions. We propose a *Human Oceans Past* research agenda to call for multi- and trans-disciplinary archaeological, historical and palaeoenvironmental/palaeoecological research to investigate: (1) when and where marine exploitation was of significance to human society; (2) how selected major socio-economic, cultural, and environmental forces variously constrained and enabled marine exploitation; and (3) what were the consequences of marine resource exploitation for societal development. We contend that this agenda will lead to a fundamental revision in our understanding of the historical role of marine resources in the development of human societies.

## Introduction

Historians have persistently undervalued past harvests of marine resources. Stories of fish as Lenten food, of whaling as a tragedy of the commons, or of fisheries as the nursery of seamen are sprinkled throughout general history books as tropes of little consequence. But what was the actual significance of marine resources to the human past? An abundant historical literature addresses single fisheries and hunts, but systematic comparative analyses are rare. Authoritative overviews such as the recent *Cambridge World History* make no more than a passing reference to the importance of marine extractions. ^1^ Archaeologists are appreciative of the role of marine life during the Paleo- and Mesolithic but after the Neolithic Revolution they too tend to focus on agricultural activities and societies. Exceptions are those that prove the rule. ^2^ Maritime history might be expected to have paid more attention to marine life, but it is often written as a story of crossings – getting people, ideas and things across a vast expanse of sea – and marine animals and habitats are lost from sight. A considerable literature focuses on human points of contact with land during marine exploitation; that is, the points of departure and arrival, cultural encounters, and maritime empires. ^3^ But the many dimensions of what occurred during fishing, hunting, and travel, including human-animal interactions and the acquisition of new and transformational knowledge about marine ecosystems and resources, are less well researched. ^4^

One reason for this oversight is perhaps a lack of readily available data, particularly when referring to long term trends and narratives of change. In the *Cambridge Economic History of Europe*, Michell observed that “One reason why the definitive history of European fishing has yet to be written is that quantitative records concerning fishing pre-1750 are few”. ^5^ Another factor may be methodological deficiencies that have encumbered the interpretation of information. However, the present ocean crisis - compounded by climate change, continuing heavy extraction, pollution of many forms (from noise to chemical), and more - has generated strong interest in the marine past to better assess its present and future. Historians and archaeologists working with ecologists have begun to establish historical baselines and thresholds of biodiversity and abundance loss, as a vital context for conservation and management. The HMAP project, running from 2000 to 2010, ^6^ was the first large-scale collaboration between historical disciplines and marine ecologists querying “what used to live in the oceans *”* and established the scale of extractions and changes to marine abundance for many species and regions, especially through the last two centuries. This research agenda continues and is evolving with the application of new methods and questions and a much deeper time horizon under the flag of the Oceans Past Initiative. ^7^

Compared to the legacy of human impacts on the marine environment, much less attention has been paid to the human contexts and human consequences of past marine resource use. Research in this field, which can be fairly labelled marine environmental history (broadly defined to include archaeology and marine historical ecology) ^8^ is still relatively undeveloped, even when compared to historical marine ecology. ^9^ Fundamental questions such as the role of seafood for historical food security, demography, and health - not to mention trade and globalisation - cannot be reliably answered with present knowledge. ^10^ Iberian, Latin-American and Southeast Asian scholars have been leaders in highlighting the gaps in our global knowledge ^11^, including a vision of the Global South. ^12^ Studies of the social and environmental forces and imperatives that varyingly drove or constrained marine exploitation, as well as the wider implications of marine resource use for economic, social, and political developments, consequently lack a solid underpinning.

In this paper, we propose a research agenda for the study of the importance of marine resources for the development of human societies in the last two millennia prior to industrialised fishing and whaling. While we recognize the importance of marine resources for earlier periods of human development, we concentrate on this period because of the relative abundance of evidence and the importance of the known marine events therein.

### The abundance of evidence for oceans past

It can be imagined that the sea washes away all traces of the past – that the history of human interaction with marine life cannot be written because of the lack of documentary and material evidence. In fact, evidence is abundant when we look with determination. It is true that material evidence for the long history of humans and the sea must be wrested from an increasingly vulnerable evidence base, impacted by sea level rise, erosion and the melting of permafrost, thereby adding urgency to the questions we ask and highlighting the short (but for the moment still open) window of access to a wealth of archaeological material that may soon be lost. ^13^ Primary documentary and visual sources may also be gathered from regional and national archives, ranging from folklore and oral histories to general and natural histories, logbooks and journals, leaflets and newspapers, laws, tithes and taxes, cargo lists, import/export and fishing statistics to maps, illustrations, paintings and many different kinds of imagery. ^14^

An example of this potential is the hunt for Arctic sea mammals. This is a history that may be thought well-known as a relentless story of a hunting down toward extirpation or near extinction until the commercial whaling moratorium of 1985. ^15^ However, abundant evidence that may modify and add cultural specificity has not yet been fully analysed and we are bound to be in for surprises. Archival data have revealed unanticipated knowledge - such as the provision of much of Britain’s 18th-century baleen by Inuit of southern Baffin Island, Canada. ^16^ Yet many research opportunities, especially for large-scale analyses, are only newly possible and remain to be realised. Adding to previous knowledge of British and American whaling in the age of sail, ^17^ one example is a recently completed data stream on British Arctic whaling, 1750–1850, detailing more than 24,000 voyages by whalers and seal hunters to the Arctic and sub-Arctic regions of the North Atlantic. ^18^ This database includes detailed information about whaling processes, whaling ground locations, the effort expended to hunt these leviathans, and the human cost in actual lives. Further documentary and visual sources may inform of practices and the processes of knowledge accumulation ( *e.g.*, by trial-and-error) and appropriation ( *e.g.*, by observation of Indigenous techniques) that enabled the hunt. ^19^ Paleoenvironmental and genomic data and modelling may reveal the ecosystem variability and processes that variously facilitated and constrained the hunt (not least by influencing species abundance and geography), ^20^ while information on prices, uses and fashions can provide insight into the economic and cultural drivers of human consumption of cetaceans. ^21^

The use of archaeological evidence (zooarchaeological, ancient DNA and stable isotopic data) to study the exploitation of Arctic sea mammals is also well developed and holds yet further potential. To offer only a selection of examples, one can employ this array of evidence and techniques in tandem with paleoenvironmental data to study climatic/sea ice impacts on potential resource availability, ^22^ serial depletions, ^23^ ecosystem cascade effects emerging from the removal of keystone species ^24^ and the complex relationships between sea-mammal hunting and social organisation. ^25^

Advances in Big Data analytics and visualisation technologies are enabling ever-more-ambitious regional to global mapping of human engagement with marine resources. ^26^ However, the use of historical and archaeological datasets without critical understanding of underlying quality and biases is an increasing problem in Big Data analysis of climate and environment. ^27^ Systematic comparative analysis is much needed as an increasing body of literature addresses single fisheries and hunts ^28^ with some (mainly archaeologically based) overviews of the ecodynamics of regions that include marine perspectives. ^29^ The use of Big Data must therefore go hand in hand with the development and application of methodological and analytical competencies. The sheer scale of potential information underlines that any future breakthrough will come as a result of a collaborative effort between archaeologists, historians, and specialists in data science. The potential results call for action, while the challenges ahead underline the need for a shared, clearly articulated research agenda.

### The Human Oceans Past agenda

A global research agenda for marine environmental history should uncover the rich and varied pathways - enabling and constraining forces and motivations, from knowledge and ideologies to demographics and environmental variability - that underlay strategies of oceanic resource use and non-use. It should also examine if, when and how barriers of knowledge, technology, and culture were overcome and with what consequences for societies and the environment. We contend that human societies diverged in their awareness and uses of marine wealth through time. Periodic “discoveries” or developing appreciation of these resources represented injections of novel wealth that potentially helped societies escape food shortages, triggered long-term socio-economic (including demographic) consequences and ecological impacts, while other societies did not apparently embark on any significant marine resource use, or incurred costs and derived benefits that were markedly unequally distributed by class, gender and more. Where they were drawn upon, marine resources were socially metabolised, i.e. valued, consumed, and energetically transformed for food, clothes, energy, health, adornment and more. In these diverse ways they played a significant yet - we contend - underappreciated role for trajectories of human demography, wealth and culture.

Economic studies indicate the importance of agricultural and maritime strategies. Regions characterised by an early Neolithisation, *i.e.,* a transition from hunter/gatherer to farming economies, enjoyed long-term economic growth, but this advantage did not endure into the modern age in any clear way. Early agriculturalisation is not a powerful predictor of early industrialisation or affluence today, it seems. ^30^ On the other hand, a recent econometric study found that “countries with access to a greater bounty of the sea — or more generally with a population descending from such areas — could benefit earlier from the new opportunities that industrialization offered, which facilitated an earlier take-off to sustained economic growth”. ^31^ The ‘Bounty of the Sea’ theory proposed to explain this divergence is that coastal regions with access to productive marine resources benefitted from a diversity of occupations that prepared the population better to take advantage of the opportunities of industrialisation. However, these studies are based on highly aggregated data of limited time-depth. The baseline study of the ‘Bounty of the Sea’ index employs FAO statistics of 1950, which are skewed towards industrialised regions. Nevertheless, the findings raise interesting questions about global historical development, which must also be set within the context of long-term negative feedbacks of industrialisation, not least for oceans. ^32^

In contrast to a positive economic assessment, ecologists perceive the impact of humans on marine life as an often inexorable trajectory of, first, targeting larger fauna, serial depletion and “fishing down” the food web. ^33^ Historical ecologists have outlined a generalised model of cultural transitions, each characterised by increasing human impact through fishing and habitat destruction. ^34^ Nevertheless, historical ecologists often describe pre-industrial harvesting technologies as sustainable. ^35^ While these perceptions have gained wide acceptance, and may often be empirically true, they rest on very patchy evidence that prevents an understanding of spatial, temporal and societal variability. Beyond important but isolated case studies, ^36^ whether pre-industrial fishers and sea mammal hunters could typically approach ( *e.g.*, given available technology) the ecological limit of their fisheries is largely unknown.

We propose, therefore, a Human Oceans Past agenda to assess the importance of marine life for human societies, beginning with the early emergence of major foci of production. Revealing this history will open a new window onto the socio-ecological dynamics and associated long-distance marine-terrestrial ecological teleconnections that have been of fundamental importance to the development of the modern world. ^37^ It will reveal historical trajectories towards sustainable and unsustainable food security and resource extraction (gathering, hunting, fishing) and the complex cultural and environmental (including climatic) processes that drove them. Such an undertaking can only take place as a collaborative effort to overcome the shortcomings of methodological nationalism, linguistic barriers, and deficiencies of funding. We believe that a global agenda will help focus the emerging field of marine environmental history around a set of bold but simple research questions that may be answered by pooling data and methods from a diverse range of historically minded disciplines, including archaeology, anthropology, ecology, geography, history, paleo-oceanography and many more.

We propose three straightforward research questions for the Human Oceans Past agenda:

1 - When and where was marine exploitation of major significance to human society?

2 - How did socio-economic, cultural, and environmental forces drive, constrain and enable marine exploitation?

3 - What were the consequences of marine exploitation for societal development and the oceans?

In the following, we venture some hypotheses and possible answers to these questions, focusing on the last two millennia, in the anticipation that these will be revised and indeed corrected in coming years as research unfolds.

### The significance of marine exploitation to human society

One pertinent contribution to marine environmental history has been the archaeological identification and conceptualization of the north-western European “fish event horizon” (FEH) of the Middle Ages, defined by a rapid and inexorable increase in demand for sea fish. ^38^ Although nuanced in terms of local manifestations (being regionally variable within ± *c.*200 years of 1000 CE), the fish event horizon (FEH) is recognisable across the northern waters of medieval Europe, from the islands of the North Atlantic to settlements of the Baltic Sea region. It is clear from a diversity of evidence, ranging from the consumption of herring in central Poland, ^39^ to the appearance of flatfish and herring bones in Ghent, ^40^ to major increases in herring and cod consumption (and a shift from freshwater/estuarine to marine flatfish consumption) in York, ^41^ to increases in sea fishing in the Orcadian archipelago of northern Scotland, ^42^ to the earliest evidence for the long-distance transport of dried cod (stockfish) from northern Norway. ^43^

This widespread and (within each region) rapid uptake of sea fishing, marine fish consumption and (incipient) fish trade was not completely unprecedented. It emerged from isolated precursors observable by the long eighth century CE or before. Among coastal settlements of Scandinavia, medieval sea-fishing traditions stretched into pre-history. ^44^ Here it was increasing scale and commercialisation that emerged as new developments during the FEH. Other regions ( *e.g.,* France, Britain and Ireland) had pre-existing traditions of trapping coastal and anadromous fish using fixed installations. ^45^ Moreover, long before the tenth century there were precocious examples of coastal settlements supplying small quantities of marine fish to proto-urban sites and/or the rural elite. ^46^ It is from these traditions that a rapid uptake of sea fishing and sea-fish consumption was possible around the turn of the first and second millennia CE.

The causes (and even existence) of the FEH have been much debated. The Medieval Climatic Anomaly centered on *c.*1000 CE may have influenced the natural abundance of important food fish such as cod (increasing their availability in Arctic Norway, for example). ^47^ However, studies of the provenance of archaeological fish bones now suggest an initially demand-driven process. The FEH was first fuelled by relatively local fishing, with widespread long-range fish trade following shortly thereafter. ^48^ Thus it has been proposed that varying (often interrelated) combinations of urbanisation, political centralisation and shifting dietary practices ( *e.g.* religious fasting practices, conspicuous consumption) were pertinent. ^49^ It is unlikely to be coincidental that many of the earliest centres of marine fish consumption outside Scandinavia were (proto-) towns and elite centres.

The inability of freshwater fisheries to sustain increasing demand was probably equally important. Hoffmann proposed that medieval freshwater fisheries were under serious pressure from habitat transformation and overfishing by the turn of the first and second millennia CE. ^50^ This hypothesis is supported by zooarchaeological evidence regarding changing proportions of fish taxa ^51^ and declines in the size of some freshwater and migratory species. ^52^ However, it has not gone unchallenged. ^53^ Orton *et al.*, for example, concluded that the FEH “predates any visible decline in deposition of freshwater fish, and hence cannot have been driven by depletion of inland fisheries as has sometimes been suggested.” ^54^ They raised a valuable chronological point, but their interpretation does not consider that high fishing intensity can maintain market supply of a declining resource until the point of economic extinction. Depletion of freshwater (and some anadromous) fish in medieval Europe is corroborated by subsequent price increases, the widespread adoption of aquaculture, and efforts by the elite to monopolise access. ^55^

The consequences of the FEH were wide-ranging in space and time. It contributed to the urban revolution of the Middle Ages, by providing a storable source of protein also suitable for Lenten fare – although based on present stable isotope evidence the contribution to whole diet may typically have been under 20%. ^56^ It integrated distant regions and underpinned the wealth and competition of both princes and places (not least the towns of the German Hanse). ^57^ Fish trade emerged on a scale to rival the better-known medieval wine trade. ^58^ Famous international products included Skåne herring and Norwegian stockfish, but they were not unique. Before the dominance of Skåne herring, an eastern English fishery was of pan-European importance. ^59^

We posit that marine developments comparable to the North European FEH have occurred elsewhere, but where and when is largely unknown. An important global research task is therefore to establish whether, where and when Marine Event Horizons (MEH) occurred globally. These MEHs might entail rapid increases in the extraction of local marine resources, the rapid uptake of storable (and hence transportable) products from increasingly distant waters, and/or the rapid spatial expansion of fishing and or hunting peoples. All three occurred during the FEH of the north-western European Middle Ages, but might elsewhere happen in differing combinations or multiple iterations. ^60^ A likely early example of an FEH is the rise of a commercial fish trade from the Black Sea that was certainly in existence as early as the sixth century BCE and linked to the rise of Greek city-states. ^61^

A number of examples outside Europe merit close attention and comparative analysis. MEHs with rapid onsets of widespread marine consumption can be thought of as episodes of innovation, not always involving a large quantity, but often with important qualitative social impacts. An increase in fishing in California’s Channel Islands with the adoption of shell fish hooks *c.*600 BCE may be understood as an MEH and a necessary precondition for a later raised level of extraction associated with the development of large sedentary settlements from the ninth century CE. ^62^ In Arctic Canada and Greenland, the rapid adoption of bowhead whale hunting around 1200 CE was likely an MEH associated with the expansion of the Thule people from Alaska to north-western Greenland within a generation. ^63^ More speculatively, one might ask whether increased reliance on marine resources, identifiable as an MEH, was initially associated with the rapid colonisation of East Polynesia (ultimately reaching Hawai‘i, Rapa Nui, and New Zealand), a spread that started in the 10th to 12th centuries CE following drought and perhaps resulting famine in Samoa and Tonga. ^64^

Further, we posit that MEHs may be followed by accelerated marine extraction (AME), a step change into big hunts and large fisheries. At their largest scale, these entailed commercial exploitation of marine resources for markets prevailing in large interconnected ( *e.g.*, imperial) systems, up to and into the globalising world. Probably the earliest example of an AME was the Atlantic-Mediterranean saltfish and garum production for Roman imperial markets around 100 BCE-500 CE. ^65^ Around 1500 CE, the discovery by European fishers of large pristine cod stocks off Newfoundland led to an acceleration in marine exploitation that delivered vast cod catches to the European market. Between 1520 and 1620, cod supplies to Europe increased nine-fold and human-per-capita consumption almost doubled. The acceleration has been labelled the “North Atlantic Fish Revolution”. ^66^ Other likely AME candidates are the 19th century fur seal hunt of New Zealand for the Canton and London markets ^67^, the rapid decimation of sea otters in the North Pacific during the 18th and 19th centuries for predominantly Asian markets, ^68^ and the right whale industry of the early modern period for a primarily European market. ^69^

Together, the MEH and AME phenomena can be understood to comprise an innovation S curve, with MEHs representing the initial discovery or adoption phase and AMEs *,* phases of exploitation of major economic and ecological significance. Both phenomena had profound socio-ecological consequences in the North Atlantic realm, yet whether, when and where similar developments happened elsewhere globally, for what species, in what environmental and societal contexts, and with what consequences, remains to be established.

So far, we have talked about marine resources in terms of extraction levels and their role as food. However, the economic and social value of marine resources in different global contexts is similarly poorly understood. One fundamental reason for this is that the role of marine wealth in human history is underappreciated. An illustration is the historiography of the early-modern Atlantic, which has been dominated by a fascination with the mineral wealth obtained by primarily Spanish expansion into Latin America. The value of gold and silver, and the role of these ‘coveted’ and ‘pivotal’ resources ^70^ in driving global trade and population movements is a recurrent theme in history, archaeology, economics and sociology. ^71^ In contrast, the economic and environmental impact of extracted Atlantic marine wealth across time remains patchily understood.

We may indicate the implications of this negligence by an example. Thirty years ago, D. B. Quinn, the historian of European Atlantic voyages of discovery, remarked that “Newfoundland, - the fish of the Banks, the inshore cod fishery, the whale fishery on its northern flank - may in the end prove to have been for Europe during the sixteenth and early seventeenth centuries as valuable a discovery as the gold and silver of the Spanish empire.” ^72^ He quoted Thomas Morton who in 1637 praised the fishing potential of New England: "The Coast aboundeth with such multitudes of Codd, that the inhabitants of New England doe dunge their grounds with Codd; and it is a commodity better than the golden mines of the Spanish Indies, for without dried codd the Spaniard, Portingal, and Italian, would not be able to vittel of a ship for the Sea" (New English Cannaan (Amsterdam, 1637), pp. 86–7). Quinn’s speculation gained little traction with historians, although Peter Pope later noted that the number of seamen involved in the fisheries far exceeded those in the Spanish silver convoys. ^73^ Recent research has established annual landings of cod in Newfoundland, ^74^ and we calculated that the value in Europe, based on the price at the port of Bilbao, equalled 70% of the value of the imports of gold and silver from the mines of America. By the 18th century the fish landings had almost doubled. ^75^ So, Quinn was right, and his comment astute: “And yet there is the strangest anomaly in the histories of the time. The conquest of the Spanish Indies is dramatic and colourful, was written about extensively at the time and ever since, while Newfoundland and its produce has remained, relatively, a backwater in world history, something to be taken for granted perhaps, but not assessed as being in any way of major significance for European development as a whole.” As Quinn suggests, the reasons for this neglect by historians may be found in the glimmer of silver and gold.

More fundamentally, however, the NorFish project identified problems of data abundance and methodology. An assessment of the scale of the fisheries demanded that the sheer wealth of documentation preserved in the French notarial archives be overcome, and strategies devised to compare these findings with the English navy records of fisheries in 120 locations. Archival studies, data curation and the development of analytical tools took several years and would have been inconceivable without large-scale funding. The result was a fundamental revision of estimated catch volumes. Historians and biologists, building on older research, have taken for granted that Newfoundland and Grand Banks catches were in the order of 200,000 metric tonnes through the early modern period. ^76^ We now calculate that in the second half of the 18th century, annual variability of catches rose to levels that were three times as large as previously thought. ^77^ The implications of making this surprising amount of protein available for a rising European population await scrutiny, as does the systematic comparison of the two Atlantic streams of wealth, southern silver and northern fish. One preliminary hypothesis to be pursued may be that while the silver caused an era of price inflation, the fish fed a growing European population and helped overcome a Malthusian trap.

Similarly, the significant early (10th to 13th century CE) ecological and socio-economic impact of marine resource extraction in Europe (fishing) and the Arctic (walrus hunting), created considerable (if sometimes short-term) wealth by capitalising upon and sometimes notably diminishing animal populations. This has been demonstrated by the Leverhulme Trust-funded Medieval Origins of Commercial Sea Fishing and Northern Journeys projects. ^78^ Analogous examples of transformative economic and ecological developments have been sketched in studies of Pacific sea otters, ^79^ bowhead and right whales. ^80^ These distinct findings provide glimpses of a full insight that only a global comparison can discover.

### The driving forces of marine exploitation

Humans are driven not by what we eat but by what we want to eat. The hunt for seafood - and other uses of marine life such as fuel, ornamentation, art, curiosity and even companionship ^81^ - has driven us far and wide, and societies have diverged in their relative usage (or non-usage) of the ocean. What factors, then, have driven, constrained and enabled marine exploitation? This is a complex question that is often left unanswered or contingent upon the preference (and disciplinary background) of the inquirer. While some see human exploitation of the sea as largely dependent on key variables such as proximity and regional variation in natural abundance, others see human agency as the primary driver. A key challenge is therefore to untangle questions of causation that until now have seemed unsolvable. The Human Oceans Past agenda may consider this problem by breaking it down into questions of knowledge, drivers, and consumption preferences.

***Knowing the ocean*.** Humans turn “Nature’s” bounty into natural resources by culturally distinct cognitive processes. Marine-human entanglements are mediated by shifting knowledge of the environment, resource extraction methods and technologies of transport that influenced mobility and settlement patterns, economics, social structures, as well as cultural transitions. ^82^ These developments depend on the expansion of societies, adaptations to local naturally and culturally diverse realities and changes, and the resulting socio-cultural interactions and new practices. ^83^ Historical inquiry should, therefore, try to identify how humans engaged with this unknown and often hostile water world and identified marine biodiversity as resources.

The knowledge and processes of observation and learning that informed and then further accrued from marine extraction are little understood, and there is no consensus on a systematic global scale. It has been variously argued that exploitation occurred ‘with limited knowledge of marine life and habitats’ ^84^; that pre-modern exploitation of marine resources was sustainable and based on ecological understanding; or conversely that early human exploitation followed a pattern of depleting higher trophic levels in what has been called a ‘sequential collapse of marine fauna’. ^85^ Knowledge exchange between points of contact and their development over time is only very patchily understood. Commonalities and differences in the relationships of humans with the ocean across time and space led to convergent or divergent ways of knowing marine ecosystems and animals. ^86^ It is sometimes assumed to have been either an asymmetrical knowledge production and transfer between Indigenous people and Europeans - for example European arrivals in the Americas or Oceania - *or* a rapid loss of traditional ecological knowledge - potentially followed by environmental mismanagement - at a colonial watershed. ^87^ Local and traditional practices were certainly sometimes ignored or even annihilated. ^88^

One major focus of a Human Oceans Past agenda must therefore be on Indigenous and external views and ways of exploitation. Medieval and early modern knowledge production about the ocean frequently resulted from encounters and confrontations, often between asymmetrical powers, and with control over resources and people as imperatives. Local knowledge and expertise were exchanged through informal and formal networks of contact between societies and imperial systems and contributed to the construction of local and global extractive regimes as much as the science and techniques behind it. ^89^ A full-scale inquiry must include processes of identification and recognition of valuable resources in distant geographies and ecosystems, learning processes and knowledge appropriation versus implementation of fishing/hunting techniques, transfer processes from places of extraction to places of consumption, and from places of Indigenous and traditional knowledge acquisition to centres of globalising knowledge. ^90^

Iberian documentary, iconographic, cartographic, and material sources for different parts of the globe, for instance, are still under-studied and in urgent need of inclusion in global histories and historiographies. ^91^ Most of these sources have not been considered by historians and many of them remain almost unknown. ^92^ Societies exploited and culturally interpreted the aquatic world in a variety of ways, ranging from resources used as food items or medicine, to those transformed into daily objects, to the creation of religious or magical icons and symbolic entities. ^93^ Marine animals have been inscribed in local legends, oral stories, letters and manuscripts, cargo lists, meal menus and pantry lists, in art and natural history treatises. ^94^ One can rescue the information retained in these diverse sources, as well as their value to peoples and individuals. Similarly, cultural heritage remains as an indication of past value and the material evidence can offer further insights to understudied regions of the world and societies.

It is a priority to conduct a systematic search in documentary and iconographic sources, oral histories and (zoo)archaeology for evidence of the historical actors that are seemingly invisible or have been neglected *but that are there* – women (and the role of gender in the exploitation and knowledge production and transfer), the role of Indigenous peoples and local societies as well as, for instance, of enslaved Africans and Native Americans in colonial contexts. The latter were not only a labour force but also had a role in marine resource access, exploitation and distribution. Most of these aspects are often underappreciated, if not forgotten, and the interaction between different individuals or societies with their more-than-human aquatic worlds did, in fact, create ‘place-based knowledge’ based on empiricism and local access to resources. ^95^ There is plenty of evidence of European dependence on local and enslaved labour in their colonial empires. But we know very little about key aspects, such as the employment of African male slaves as harpooners in shore-based whaling in Brazil (and maybe in the Caribbean), or about local ecological knowledge and access to empirical understandings of seasonal species presence, distribution of resources and fishing techniques, both in South America and Africa. ^96^ Moreover, the role played by women in activities and particular tasks related to fishing, hunting and gathering, including their role in informal but non-trivial markets and local societies, need to be brought to light. ^97^

Let us consider an example of how colonial and imperial systems reflected the uses of marine life, of how local and indigenous systems evolved and were kept or annihilated, and how both interacted and impacted one another. In every river and coastal area of Central and South America, manatees (also known as sea cows or ox-fish) have historically been used as a valuable resource by indigenous populations. They were caught by harpoons, nets, ropes, traps and even remoras (“fishing fish”), depending on the geographic region and the Indigenous community, and probably also according to the habitat and size of each individual. In some societies, women (as partners to the fishermen) were a vital element of the search and hunt for the animals, taking an active role in all the processes, not just in the butchering and distribution of the parts. The arrival of the first Europeans to the islands and coasts of the Americas intensified the impact of human populations on these animals. It also reinforced the European understanding of aquatic bodies as a birthplace of life, diversity and abundance; even more so in these lands, where this abundance was apparently endless. ^98^ This idea of never-ending biodiversity and super-abundance is clear in European knowledge production alongside details of the anatomy, ecology, behaviour and the different uses of the animal. Documentary and iconographic sources of the sixteenth and seventeenth centuries show us that manatees were used as food and for medicinal purposes, to manufacture tools or even as pets, across their distribution range. The animal was inserted in the annals of natural history and philosophy, and of art and literature. ^99^ Alongside manatees, other aquatic megafauna species, such as turtles and coastal and river fish were being heavily exploited upon European colonisation and habitat transformation.

A similar pattern was found in West and East Africa where manatees and dugongs, respectively, were hunted and used by local societies and became a major target of interest upon European arrival and colonisation. ^100^ Europeans learnt about and experienced these animals and other nonhuman elements of the tropical regions through direct contact with local societies of Asia, Africa, the Caribbean and South America, and empirical knowledge was brought to Europe via transatlantic economic or scientific communication. ^101^

The history of aquatic-human interaction may reveal moments of protagonism by the Indigenous peoples of the Americas in pre-contact and early modern times, of local and traditional groups across the African shores as well as across the Indian Ocean. This long-term experience may also illuminate the agency of the animals themselves. The Human Oceans Past agenda should contribute to placing the animals (as resources and as symbols) and their ecosystem at the centre of the discussion about the environmental and sociocultural contexts and interactions of societies in pre-modern contexts. The example of Sirenian species highlights the paradoxical relation of aesthetic and emotional appreciation and utilitarian and commercial use, as well as patterns of convergence in the use of similar aquatic resources. These issues of knowledge production and exchange are pertinent globally, and increasingly a subject of study in the context of growing appreciation of the importance of traditional ecological knowledge in resource exploitation and management. ^102^

***Integrating understandings of natural and anthropogenic drivers*.** Marked variability in extractions is often a defining feature of long records of marine exploitation. ^103^ Key related questions thus concern the relative contribution of natural versus human factors to this, and indeed whether extractions were for much of history (considering prevailing technologies and low population-driven demand) so far below most ecosystem limits, or extractions so heavily influenced by human factors ( *e.g.* conflict or piracy impacting the fisheries), such that little of the observed variability can be of natural origin. ^104^

The case of the Swedish Bohuslän herring phenomenon is instructive of possible interactions and feedbacks between natural and human drivers of marine exploitation. Off the Bohuslän coast, north of present-day Gothenburg, great herring shoals have appeared on a quasi-centennial time scale (c.1556–1590, 1747–1808, 1877–1906), persisting for several decades at a time, generating huge wealth and attracting tens of thousands of migrant workers in peak times. ^105^ Historians thus recognize the Bohuslän herring fishery as a major factor in economic and social change in Western Sweden, as well as an important element in shaping European fish markets. ^106^ In this we may posit that the rapid and heavy exploitation upon each onset of the phenomenon must have depended upon an acute environmental awareness, alongside sufficient technological prowess and a sufficiently mobile labour pool to power its exploitation. A market sufficiently integrated to facilitate transport and sale, and a population sufficient to demand a notable supply, must also have prevailed to drive exploitation that was clearly beyond that needed for local subsistence. The scale of exploitation and associated employment, as well as the eventual adaptation of the resource away from subsistence in order to serve new technologies and markets, is highlighted by Sahrhage and Lundbeck, who note that “around 1760 a method for the production of herring train oil was discovered and all herring, except the leanest, were then utilised [for this, with…] almost 500 oil factories along the Bohuslän coast” in the mid-1790s. ^107^ Given the capital and labor invested in exploiting the phenomenon, its periodic appearance and (often sudden) decline may have triggered profound economic, social, cultural and political effects and put societal resilience and the adaptability of peoples and economies to the test. Indeed, the study of such "cyclonic” volatility in natural resource availability provides an important opportunity to address longstanding debates about the societal benefits versus negatives of marine and other natural resource abundance. ^108^

The Bohuslän phenomenon is renowned in marine science as an outstanding example of a centennial-scale oscillation in pelagic species geography and abundance, the onset and cessation of which is driven by complex environmental (and perhaps even socioeconomic) factors yet to be fully understood. ^109^ In this respect, we note that marked pelagic ecosystem variability is known elsewhere, for example in association with the California Current, Humboldt Current, the Kuroshio Current (and its extension into the East Korean Sea/Sea of Japan, the Tsushima Current), and in the English Channel. These provide opportunities for comparative studies of socioeconomic impacts and other human responses, as well as the natural drivers of this variability and the ecosystem consequences of exploitation.

Relevant to the above, most know that the famed “year without a summer” followed the great 1815 eruption of Tambora, Indonesia. ^110^ Few know, by contrast, that 1816 was also known as the “mackerel year” for the supernormal catch volumes that this eruption likely precipitated in the Gulf of Maine. This occurred at least partly as a consequence of terrestrial crop failures and incipient famine that pressed fishers, already faced with insufficient alewife runs, to heavily exploit the mackerel population that seasonally migrated into the region (both species having already been adversely affected by the volcanically induced colder waters). This shift to greater exploitation of mackerel and away from the alewife persisted, moreover, despite the recovery of alewife populations and terrestrial agriculture in the following years. Human response to this singular extreme climatic event can thus be credited with a long-term alteration to this ecosystem and its human entanglements. ^111^

All major explosive eruptions hold the potential to induce simultaneous or closely timed impacts that may range from the positive to the adverse, resulting in different net outcomes in terms of human benefit and behaviour, ecosystem productivity and their interactions, as mediated by the characteristics of the specific ecosystems and societies in question. Such eruptions, the dating and magnitude of which are now known with high confidence using polar ice-core records of volcanic sulphate deposition for the past 2500 years, ^112^ are also increasingly recognized as the dominant driver of severe and abrupt climatic changes on interannual to multi-decadal time-scales over past centuries and millennia. ^113^ Beyond potential impacts on ocean productivity from direct fertilisation by nutrient-rich volcanic fallout over water, ^114^ volcanic climatic impacts can also be of potentially net positive impact for pelagic species in regions such as the North Sea. Diverse evidence from medieval accounts of extreme weather to contemporary climate modelling associates major eruptions with severe cooling in the greater northeast Atlantic, including the North Sea. ^115^ Here, colder surface waters result in a less stratified water column and a consequently greater mixing between upper and lower level waters, the latter relatively nutrient-rich. This promotes increased abundance of the zooplankton upon which North Sea herring feed, ^116^ thereby representing a further potential forcing of the Bohuslän phenomenon that is yet to receive full scrutiny. With multiple volcanic eruptions now known and well dated in ice-core-based reconstructions, they provide powerful comparative tests of socio-ecological response to sudden environmental shocks between regions. In the Sea of Japan/East Korean Sea, an environment not dissimilar to the North Sea, the indigenous Ainu people who presently inhabit its northern extent (near Sakhalin Island) describe a sea god called Repun who appeared to deliver a bountiful fish catch when the hunt on land was poor. ^117^ As well as hinting at the importance of recourse to marine resources to prevent subsistence crises, it is tempting to see the contrasting failure of the hunt on land at a time of apparent success at sea as a further potential reflection of past volcanic climatic and ecosystem impacts. A search for further parallels should prove insightful.

With increasing volumes of data from paleoclimatic reconstructions, climate and ecosystem model output, social and economic data from written and archaeological sources, Big Data approaches now allow for holistic analyses of data *across key thresholds* in global environmental, demographic and societal change and development. ^118^ Large temporally extensive datasets also enable the study of marine resource use before and after the colonial watershed of the early modern period (1500–1800), before and after the medieval Eurasian expansion and its associated FEH (800–1200), and before and after the empires of the early Common Era (100 BCE-500 CE). Crucially, many such chronologies span major regional to potentially global climatic phenomena that likely influenced both marine ecosystems and the societies exploiting them, not least the Roman Warm Period, Late Antique Little Ice Age, Medieval Climate Anomaly and Little Ice Age – prior to fossil-fuel-induced global warming.

***Consumption*.** Extraction industries are often written from the production side as an output-driven history. However, demand was what enticed mariners to undertake long and dangerous travels – or indeed not utilise the seas at all. Consumption history reveals the environmental and cultural factors impacting the choice of food from the high dependence on marine resources in the Arctic ^119^ to the terrestrial diet depended upon by 19th-century European settlers in Australia, ^120^ while other cultures revered great mammals to the point of tabooing the hunt. ^121^ Some societies abandoned the sea almost completely, exemplified by the striking loss of boat technology in the Canaries ^122^ or Rapa Nui after the islands’ first settlement.

The long-range impact of consumer demand is known to have dramatically reduced abundance and indeed driven species to near-extinction levels. Examples include the above-mentioned New Zealand fur seal trade through London and Canton to serve a primarily Chinese fashion, ^123^ and the depletion of oyster banks across the North Atlantic to feed the needs of restaurants. ^124^ The oceans became arenas of big hunts that decimated whales and seals to light the streets of the world ^125^ or to ameliorate food crises ^126^. However, consumption history is surprisingly uncharted. Estimates of global seafood consumption only go back to 1961 ( FAO fishery statistics, accessed 27 November 2020).

There are great methodological challenges to studying consumption history both as regards the archaeological and documentary evidence. As introduced above, for example, it is often assumed that European medieval fish consumption was partly determined by Catholic prescriptions of Lent – and conversely that early modern Northern Europe saw a religiously driven decline in seafood consumption. Recent empirical studies have questioned this widespread hypothesis. Western European annual per capita consumption of cod and herring increased by 50% between 1525 and 1625. ^127^ Consumption decreased during the 17th-century military and climate crisis but increased again through the 18th century when long-distance cod fisheries boomed. This trajectory indicates that the growth of the Grand Banks and Newfoundland fishery may have played a significant role in human demographic growth by providing increased food security during the early modern period - and that assumptions about the dietary effect of the Protestant Reformation need to be revisited.

Archaeology, which revealed the FEH, is unfortunately less helpful for now as regards early modern seafood consumption. Post-medieval zooarchaeological evidence has suffered from the impacts of emerging civic refuse disposal practices in the past, and both limited research interest and the disturbance of recent archaeological strata by modern development in the present. ^128^ The use of stable isotope analysis of human bone to infer changes in marine protein consumption from medieval to modern times is also complicated - due to increased global mobility (introducing the variable of differing ecosystem baselines) and the globalisation of crops such as sugarcane, millet and maize, which have a C _4_ photosynthesis pathway that mimics the isotopic signature of marine fish. Progress is being made in the archaeological evaluation of post-medieval diets, using multi-proxy methods, but much remains to be done. ^129^

As noted above, food was not the only aspect of consumer demand to have major implications for human interactions with marine fauna. Oil and baleen were also of great importance in specific times and places. Right whales provide one example of a global story still to be written. These whales, closely related species of the genus *Eubalaena*, are migratory, highly coastal and highly accessible, with a global distribution, and their interactions with different societies have been long-term and often intense, including the exploitation of strandings and active whaling. The latter led to early population extirpations. Right whale numbers today are very low, and they face the risk of extinction: *Eubalaena glacialis* is already extirpated in the eastern North Atlantic, and only *c.*250 mature individuals survive in total. Attempts have been made to estimate past abundance, ^130^ but past studies lack global coverage as well as the integration of cultural, economic, and ecological drivers - at local, regional and global levels - and consequences of long-term exploitation. Northeast Atlantic right whales were the main target of whaling at least since medieval times in the Basque Country and probably also in Portugal. Their continued and well-organised hunt put unprecedented pressure on natural populations of migrating mothers and calves and, as importantly, influenced the transfer of practices from medieval Europe to the early modern colonial settlements of North and South America. ^131^ Studying this taxon has the potential to reveal local practices of shore-based whaling in Europe and in extra-European regions.

Much is also still to be understood in the global South pre-European contact. Addressing the exploitation and use of right whales and other aquatic mammals across different areas of their past distribution will open our minds to traditional and indigenous practices ( *e.g.,* in the American continent), and to never-studied populations. Examples include Southwest and Southeast Africa, where later major foci of colonial whaling occurred in Namibia and Mozambique in direct relation with historically known whale breeding grounds ^132^. Using archaeology, it is also possible to identify and study forgotten historical populations of right whales, such as in the Mediterranean Sea. ^133^ In fact, their presence along the shores of the Roman Empire raises the hypothesis that they may have formed the basis of a forgotten whaling industry of the early first millennium CE.

The evolution of regional and global marketplaces or metropoles like Paris, London, Lisbon, New York, Hong Kong and Tokyo played a significant role in driving demand and distributing marine wealth. Urban nodes provided global marine biodiversity to consumers by trade, human migration, knowledge, cultural exchange, and financial flows. ^134^ At the same time, the markets had widespread ecological ramifications by driving up fishing pressure and increasing access to marine biodiversity. Pioneering studies have been conducted on the archaeological and documentary evidence of single cities ^135^ but the overall picture is still largely unknown. One should raise simple but important questions such as: Which species were sold? How much represented local resources, versus fish from further afield, including transoceanic? How much of the fish was fresh (always much more expensive) and how much was dried/salted? The answers will reveal changes in consumption, logistics and profits and provide the basis for wider analyses of pre-modern perceptions of the natural world. Consumption history has the potential to reveal the ebb and flow of networks and the market nodes that partly created them.

## Discussion: The consequences of marine exploitation for societies and for the oceans

We contend that the role of marine resources has been systematically underestimated in a historiography that has (by relative weight of publications) focused on terrestrial resources. The long-term development and relative success of marine resource use depended on how societies came to know of these resources and overcome the many technological, environmental, and cultural obstacles to their access, including the potential opportunity costs of pursuing marine resources relative to terrestrial resources. In effect, how marine resources were socially metabolised to become marine wealth. Societies consumed, traded, and energetically transformed marine resources for food, clothes, energy, health and adornment, in diverse ways that have played a significant role for trajectories of human demography, wealth and culture. Geographical, technological, economic and cultural pathways conditioned societies in distinct ways and could create deep linkages between coast and hinterland. ^136^ Innovative deep charts based on GIS technology are beginning to visualise the processes, patterns and networks involved, revealing patterns of perception and knowledge. ^137^ One can map the spatial dimensions of marine metabolisation and financing to examine how marine-resource use created or contributed to path dependencies that led to certain historical geographical outcomes, asking related questions such as whether and how areas far removed from the sea benefitted from marine resource extraction.

Marine wealth was probably much more widely distributed than land wealth. While agricultural estates and mineral wealth tended towards a concentration in the hands of an elite and left conspicuous testimony of consumption, marine wealth was likely to be distributed in a wide network of middling merchants and investors. One reason for the conspicuousness of mineral wealth such as that from the Latin American silver mines may be that the riches of the mines were concentrated in the hands of an elite who sponsored magnificent art and architecture, whereas the wealth of the fishery was more widely distributed among many agents, from fishermen to merchants to financiers. The wider distribution of wealth in the fisheries has given rise to conflicting theories of egalitarian social fabrics of fishing communities. ^138^ How these communities responded to the potential for sudden peaks and troughs in marine resource availability also remains an important question. The impacts of such "cyclonic volatility" in natural resource abundance could be profound, with historical cases such as the Bohuslän phenomena providing valuable case-studies of societal adaptation and resilience, and relatedly who ultimately benefitted from natural resources. ^139^

Ocean resources have generated immense geopolitical interest, and conflicts over access and domination left a deep and conflicted legacy. Therefore, the outputs from marine extraction not only drove the global economy but played a major role in politics and society. Voyages of discovery and the development of cartography display the role of marine resources through abundant depictions and descriptions that lured potential profiteers. ^140^ It must be a priority to map and explain overlapping local, indigenous, national, colonial and imperial interests in the exploitation of sea resources, and moments of confrontation over access and trade, such as the conflict between Portugal (in Brazil) and England (in America) over offshore sperm whale hunting in the South Atlantic in the late 18th century. ^141^ This conflict resulted in the discovery of the Brazil Banks and other hotspot areas as a legacy to offshore whaling in the centuries to come. In this case, whales were pawns in geopolitical disputes and a driving force in the construction of many nations. In many other cases, sirenians, turtles, sharks, seals, walruses, different species of fish, and other marine living resources were the centre of appropriation, contacts or conflicts, in short, of historical entanglements between humans and the ocean.

In sum, we propose the Human Oceans Past agenda to encourage scholars of all fields to address a set of simple but essential questions that will enhance our understanding of how marine extraction drove (and was in turn driven by) global economic and ecological change, while also playing a major role in politics and societies. To make decisive advances forward in understanding the role of the sea in human societies we need a definitive methodological step change in the integration of humanities and natural science. Pursuing the Human Oceans Past agenda will require such an integration as well as introduce much-needed chronological depth that can better address the urgent and interlinked societal and environmental issues faced across the globe, through the understanding of past commonalities and divergences in the use and perceptions of the oceans.

## Data Availability

No data are associated with this article.

## Footnotes

^1^ Marks, Robert B. “Exhausting the Earth”: Environment and History in the Early Modern World. In *The Construction of a Global World, 1400–1800 CE*. *Foundations*, edited by Jerry H. Bentley, Sanjay Subrahmanyam and Merry E. Weisner-Hanks. *The Cambridge Word History*, 29–53. Cambridge: Cambridge University Press, 2015.

^2^ Rick, T.C., Erlandson, J.M., *Human Impacts on Ancient Marine Ecosystems: A Global Perspective*. Berkeley: University of California Press (2008). Barrett, J.H., Orton, D.C., *Cod and Herring: The Archaeology and History of Medieval Sea Fishing.* Oxford: Oxbow Books (2016) (hereafter *Cod and Herring*). Fagan, B., *Fishing: How the Sea Fed Civilization.* New Haven: Yale University Press (2017). Hurk, Y.van den., *On the Hunt for Medieval Whales: Zooarchaeological, Historical, and Social Perspectives on Cetacean Exploitation in Medieval Northern and Western Europe*. Oxford: BAR Publishing (2020).

^3^ Butel, P. *Histoire de l’Atlantique de l’Antiquité à nos jours.* Paris, Perrin (1997). Steinberg, P.E., *The Social Construction of the Ocean*. Cambridge: Cambridge University Press (2001). Grafe, R., Turning maritime history into global history: some conclusions from the impact of globalization in early modern Spain, in *Maritime History as Global History, ed.* M. Fusaro & A. Polónia. Liverpool: Liverpool University Press (2010), 249–266. Armstrong, C. & Chmielewski, L. M. (eds.), *The Atlantic Experience: Peoples, Places, Ideas*. London: Palgrave Macmillan (2013). Abulafia, D., *The Boundless Sea. A human history of the oceans*. London: Allen Lane (2019). Strootman, R., van den Eijnde, F. & van Wijk, R., *Empires of the Sea. Maritime Power Networks in World History*. Leiden: Brill (2019).

^4^ Heidbrink, I., Whaling, fisheries and marine environmental history in the International Journal of Maritime History, *International Journal of Maritime History* 26, nº1 (2014): 117–122.

^5^ Michell, A R., The European Fisheries in Early Modern History. In *The Cambridge Economic History of Europe Volume 5: The Economic Organization of Early Modern Europe*, 133–84. Cambridge: Cambridge University Press (1977).

^6^ Holm, P., Marboe, A. H., Poulsen, B. & MacKenzie, B. R., Marine Animal Populations. A New Look Back in Time. In A. D. MacIntyre (ed.), *Life in the World's Oceans: Diversity, Distribution, and Abundance* (pp. 3–23). Oxford: Blackwell (2010).

^7^ Holm, P. (2003). History of marine animal populations: a global research program of the Census of marine life. *Oceanologica Acta* 25: 207–211. Schwerdtner Manez, K., P. Holm, L. K. Blight, M. Coll, A. MacDiarmid, H. Ojaveer, B. Poulsen and M. Tull,. The future of the oceans past: towards a global marine historical research initiative, *PLoS One* 9, 7 (2014): e101466.

^8^ Holm, P., T. D. Smith and D. J. Starkey (ed.), *The Exploited Seas: New Directions for Marine Environmental History*. Research in maritime history. St. John's, Newfoundland, International Maritime Economic History Association (2001). Bolster, W. J., *The Mortal Sea: Fishing the Atlantic in the Age of Sail*. Cambridge, Harvard University Press (2013). Máñez, K.S. & Poulsen, B. (eds), *Perspectives on Oceans Past. A Handbook on Marine Environmental History*. Dordrecht: Springer Netherlands (2016).

^9^ Thurstan, R. The potential of historical ecology to aid understanding of human-ocean interactions throughout the Anthropocene. *Journal of Fish Biology* (2022), 1–14.

^10^ Fitzhugh, B., Virginia L. Butler, Kristine M. Bovy, Michael A. Etnier, Human ecodynamics: A perspective for the study of long-term change in socio-ecological systems. *Journal of Archaeological Science: Reports* 23 (2018): 1077–94. Holm, P. SDG 14 - exploiting and managing the alien and unseen world below water. In: Before the UN Sustainable Development Goals, Martin Gutmann & Daniel Gorman (eds.), Oxford University Press (2022).

^11^ Christensen, J. & Tull, M., *Historical Perspectives of Fisheries Exploitation in the Indo-Pacific*. MARE Publication Series 12. Dordrecht: Springer Netherlands (2014). Guimarães, P. & Amorim, I. A História Ambiental em Portugal: A emergência de um novo campo historiográfico. *Revista Internacional de Ciências Sociais* 35 (2016), 47–58. Soluri, J., Leal, C. & Pádua, J.A. (Eds.), *A Living Past: Environmental Histories of Modern Latin America*. New York: Berghahn (2018). Cariño, M. (Dir.) Nuestro Mar: Historia ambiental del golfo de California (siglos XVI-XXI). Editorial Comares, Granada (2021).

^12^ Santos, A.O. Ocean Narratives: Fluxes of commodities across the Pacific in the contemporary age. In: *East Asia, Latin America, and the Decolonization of Transpacific Studies, Historical and Cultural Interconnections between Latin America and Asia*, C. Olivieri & J. Serrano-Muñoz (eds.). Springer Nature, Switzerland (2022).

^13^ Orton, D.C., Archaeology as a Tool for Understanding Past Marine Resource Use and Its Impact. In Kathleen Schwerdtner Máñez & Bo Poulsen (eds.). *Perspectives on Oceans Past. A Handbook on Marine Environmental History*. Dordrecht: Springer Netherlands (2016), 47–70. Barrett, J.H., “An environmental (pre)history of European fishing: Past and future archaeological contributions to sustainable fisheries.” *Journal of Fish Biology* (2019), 1–12. Hollesen, J., Callanan, M., Dawson, T., Fenger-Nielsen, R., Friesen, T.M., Jensen, A.M., Markham, A., Martens, V.V., Pitulko, V.V., Rockman, M., “Climate change and the deteriorating archaeological and environmental archives of the Arctic,” *Antiquity* 92 (2018), 573–586.

^14^ Poulsen, B., Human Archives: Historians’ Methodologies and Past Marine Resource Use. In Kathleen Schwerdtner Máñez & Bo Poulsen (eds.). *Perspectives on Oceans Past. A Handbook on Marine Environmental History*. Dordrecht: Springer Netherlands (2016), 71–88. Vieira, N., “A baleação portuguesa do Atlântico Sul na época moderna: Uma análise integrada em história ambiental marinha”. In Wellington Castellucci Junior & Daniel Quiroz (Orgs.). *Baleeiros do Sul II. Antropologia e História da Indústria Baleeira nas Costas Sul-americanas*. Salvador, Editora da Universidade do Estado da Bahia (2018a), 85–102. Vieira, N., A Comparative Approach to Historical Whaling Techniques: Transfer of Knowledge in the 17th century from the Biscay to Brazil. In Amélia Polónia, Fabiano Bracht, Gisele C. Conceição & Monique Palma (Eds/ Coord.). *Cross-cultural Exchange and the Circulation of Knowledge in the First Global Age*. 1. ed. Porto, CITCEM/Edições Afrontamento (2018b), 125–143. Roque, A.C., Towards a scientific approach of nature: Looking at Southern Africa biodiversity throughout the 16 ^th^ century Portuguese records of marine fauna. In Amélia Polónia, Fabiano Bracht, Gisele Conceição & Monique Palma (eds.). *Cross-cultural exchange and the circulation of knowledge in the first global age*. Porto: CITCEM-Edições Afrontamento (2018), 75–102. Thurstan, R. H. (2022). The potential of historical ecology to aid understanding of human–ocean interactions throughout the Anthropocene. *Journal of Fish Biology*, Early View. https://doi.org/10.1111/jfb.15000

^15^ *E.g*., Kruse, F., Catching up: The state and potential of historical catch data from Svalbard in the European Arctic, *Polar Record* 53 (2017), 520–533. Clements, C. F., Blanchard, J. L., Nash, K. L., Hindell, M. A., & Ozgul, A. (2017). Body size shifts and early warning signals precede the historic collapse of whale stocks. *Nature Ecology & Evolution*, 1, 0188. https://doi.org/10.1038/s41559-017-0188
Frasier, B. A., Springate, L., Frasier, T. R., Brewington, S., Carruthers, M., Edvardsson, R., Harrison, R., Kitchener, A. C., Mainland, I., & Szabo, V. E. (2022). Genetic examination of historical North Atlantic right whale (Eubalaena glacialis) bone specimens from the eastern North Atlantic: Insights into species history, transoceanic population structure, and genetic diversity. *Marine Mammal Science*, Early View. https://doi.org/10.1111/mms.12916

^16^ Barr, W., The eighteenth century trade between the ships of the Hudson’s Bay Company and the Hudson Strait Inuit. *Arctic* 47 (1994), 236– 246.

^17^ e.g. Smith, T., Reeves, R., Josephson, E., Lund, J., Spatial and Seasonal Distribution of American Whaling and Whales in the Age of Sail, *PLoS ONE* 7 (2012), e34905. Chatwin, D., Findings from an analysis of data in the British southern whale fishery (1775–1859) datasets. *The Great Circle* 40 (2018), 28–49.

^18^ Molloy, D. & Stonehouse, B., British Arctic Whaling Dataset 1750–1850. Dublin: TCD/OPI (2019). Supporting documentation: Molloy, D., Stonehouse, B. and Nicholls, J., British Arctic Whaling Dataset 1750–1850, Supporting Documentation. Dublin (2019): TCD/OPI Internet resource: http://oceanspast.org/baw.

^19^ As an example of knowledge transfer in whaling see Barkham, Selma H., The Basque Whaling Establishments in Labrador 1536–1632 —A Summary. *Arctic*, 37 (4) (1984), 515–519. Martjin, C.A., Barkham, S & Barkham, M.M., The Whalers on the 1546 Desceliers Map, Seen through the Eyes of Different Beholders. Research Note (2019) https://journals.lib.unb.ca/index.php/NFLDS/article/download/151/258?inline=1. For a case of knowledge appropriation, regarding manatee hunting in colonial Americas, see Vieira, N. & Brito, C., Brazilian manatees (re)discovered: Early modern accounts reflecting the overexploitation of aquatic resources and the emergence of conservation concerns. *The International Journal of Maritime History* 29, nº2 (2017), 513–529.

^20^ Cabrera, A. A., Schall, E., Bérubé, M., Anderwald, P., Bachmann, L., Berrow, S., Best, P. B., Clapham, P. J., Cunha, H. A., Rosa, L. D., Dias, C., Findlay, K. P., Haug, T., Heide-Jørgensen, M. P., Hoelzel, A. R., Kovacs, K. M., Landry, S., Larsen, F., Lopes, X. M., Lydersen, C., Mattila, D. K., Oosting, T., III, R. M. P., Papetti, C., Paspati, A., Pastene, L. A., Prieto, R., Ramp, C., Robbins, J., Sears, R., Secchi, E. R., Silva, M. A., Simon, M., Víkingsson, G., Wiig, Ø., Øien, N., & Palsbøll, P. J. (2022). Strong and lasting impacts of past global warming on baleen whales and their prey. Global Change Biology, 28, 2657–2677. https://doi.org/10.1111/gcb.16085.

^21^ *E.g.*, Schoemaker, N., Whale Meat in American History. *Environmental History* 10, nº2 (2005), 269–294.

^22^ Ogilvie, A.E.J., Woollett, J.M., Smiarowski, K., Arneborg, J., Troelstra, S., Kuijpers, A., Pálsdóttir, A., McGovern, T.H., Seals and sea ice in medieval Greenland. *Journal of the North Atlantic* 2 (2009), 60–81. Szpak, P., Savelle, J.M., Conolly, J., Richards, M.P., Variation in late Holocene marine environments in the Canadian Arctic Archipelago: Evidence from ringed seal bone collagen stable isotope compositions. *Quaternary Science Reviews* 211 (2019), 136–155.

^23^ Barrett, J.H., Boessenkool, S., Kneale, C.J., O’Connell, T.C., Star, B., Ecological globalisation, serial depletion and the medieval trade of walrus rostra. *Quaternary Science Reviews* 229 (2020), 106122.

^24^ Simenstad, C.A., Estes, J.A., Kenyon, K.W., Aleuts, sea otters, and alternate stable-state communities. *Science* 200 (1978), 403–411. Hacquebord, L., Three centuries of whaling and walrus hunting in Svalbard and its impact on the Arctic ecosystem. *Environment and History* 7 (2001), 169–185. Estes, J. A., Burdin, A., & Doak, D. F. (2016). Sea otters, kelp forests, and the extinction of Steller’s sea cow. *PNAS*, 113, 880–885. https://doi.org/10.1073/pnas.1502552112

^25^ *E.g.,* Gotfredsen, A.B., Appelt, M., Hastrup, K., Walrus history around the North Water: Human–animal relations in a long-term perspective. *Ambio* 47 (2018), 193–212; Mason, O.K., Rasic, J.T., Walrusing, whaling and the origins of the Old Bering Sea culture. *World Archaeology* 51 (2019), 454–483.

^26^ Travis, C. & Holm, P., *The Digital Environmental Humanities—What Is It and Why Do We Need It? the NorFish Project and Smart City Lifeworlds*. The Digital Arts and Humanities. Switzerland, Springer International Publishing (2016).

^27^ Van Bavel, B. J. P., Curtis, D. R., Hannaford, M. J., Moatsos, M., Roosen, J. & Soens, T., Climate and society in long‐term perspective: Opportunities and pitfalls in the use of historical datasets. *WIREs Climate Change* 10, nº6 (2019), e611.

^28^ Gibson, J.R., *Otter Skins, Boston Ships and China Goods: The Maritime Fur Trade of the Northwest Coast, 1785–1841*, University of Washington Press, Seattle (1992). Barrett, J.H., Locker, A.M., Roberts, C.M., The origin of intensive marine fishing in medieval Europe: The English evidence, *Proceedings of the Royal Society* B 271 (2004), 2417–2421. McKechnie, I., Lepofsky, D., Moss, M.L., Butler, V.L., Orchard, T.J., Coupland, G., Foster, F., Caldwell, M., Lertzman, K., Archaeological data provide alternative hypotheses on Pacific herring (Clupea pallasii) distribution, abundance, and variability, *PNAS* 111 (2014), E807-E816. Pope, P., *Fish into wine: The Newfoundland plantation in the seventeenth century*. Chapel Hill, University of North Carolina Press (2004). Butcher, J.G., *The Closing of the Frontier: A History of the Marine Fisheries of Southeast Asia, c.1850–2000*. Singapore: Institute of Southeast Asian Studies (2004). Bolster, W.J., *The Mortal Sea: Fishing the Atlantic in the Age of Sail*. Cambridge: Harvard University Press (2013). West, C. F., Etnier, M. A., Barbeaux, S., Partlow, M. A., & Orlov, A. M. (2020). Size distribution of Pacific cod (Gadus macrocephalus) in the North Pacific Ocean over 6 millennia. *Quaternary Research*, First View. https://doi.org/10.1017/qua.2020.70. Reeder-Myers, L., Braje, T. J., Hofman, C. A., Smith, E. A. E., Garland, C. J., Grone, M., Hadden, C. S., Hatch, M., Hunt, T., Kelley, A., LeFebvre, M. J., Lockman, M., McKechnie, I., McNiven, I. J., Newsom, B., Pluckhahn, T., Sanchez, G., Schwadron, M., Smith, K. Y., Smith, T., Spiess, A., Tayac, G., Thompson, V. D., Vollman, T., Weitzel, E. M., & Rick, T. C. (2022). Indigenous oyster fisheries persisted for millennia and should inform future management. *Nature Communications*, 13, 2383. https://doi.org/10.1038/s41467-022-29818-z.

^29^ VanDerwarker, A.M., *Farming, Hunting & Fishing in the Olmec World*. Austin, University of Texas Press (2006). Harrison, R. & Maher, R.A., *Human Ecodynamics in the North Atlantic - A Collaborative Model of Humans and Nature through Space and Time*. USA: Lexington Books (2014). Hambrecht, G., Feeley, F., Smiarowski, K., Hicks, M., Harrison, R., Brewington, S., . . . Gibbons, K., A millennium of Icelandic archaeological fish data examined against marine climate records. *Quaternary Research*, First View (2019) doi: https://doi.org/10.1017/qua.2019.1035. Braje, T.J., Rick, T.C. (Eds.), *Human Impacts on Seals, Sea Lions, and Sea Otters: Integrating Archaeology and Ecology in the Northeast Pacific*, University of California Press, Oakland, CA (2011). McKechnie, I., Moss, M.L., Meta-analysis in zooarchaeology expands perspectives on Indigenous fisheries of the Northwest Coast of North America, *Journal of Archaeological Science: Report*s 8 (2016), 470–485; Mason, O.K., Rasic, J.T., Walrusing, whaling and the origins of the Old Bering Sea culture, *World Archaeology* 51 (2019), 454–483.

^30^ Olsson, O., & Paik, C., A Western reversal since the Neolithic? The long-run impact of early agriculture. *The Journal of Economic History,* 80, nº1 (2020), 100–135. Litina, A., Natural land productivity, cooperation and comparative development. *Journal of Economic Growth* 21 (2016), 351–408.

^31^ Dalgaard, C.-J., Knudsen, A.S.B. & Selaya, P., “The bounty of the sea and long-run development”. *Journal of Economic Growth* 25, nº 3 (2020), 259–295.

^32^ Doney, S.C., The growing human footprint on coastal and open-ocean biogeochemistry, *Science* 328 (2010), 1512–1516. Duarte, C. M., Agusti, S., Barbier, E., Britten, G. L., Castilla, J. C., Gattuso, J.-P., Fulweiler, R. W., Hughes, T. P., Knowlton, N., Lovelock, C. E., Lotze, H. K., Predragovic, M., Poloczanska, E., Roberts, C., & Worm, B. (2020). Rebuilding marine life. Nature, 580, 39–51. https://doi.org/10.1038/s41586-020-2146-7.

^33^ Bearzi, G., E. Politi, A. S. & Azzellino, A., Prey depletion caused by overfishing and the decline of marine megafauna in eastern Ionian Sea coastal waters (central Mediterranean). *Biol Conserv* 127 (2006), 373–382.

^34^ Jackson, J.B.C. *et al.*, Historical Overfishing and the Recent Collapse of Coastal Ecosystems. *Science* 293 (2001), 629–638. Pitcher, T. J., & Lam, M. E. (2015). Fish commoditization and the historical origins of catching fish for profit. *Maritime Studies*, 14:2
10.1186/s40152-40014-40014-40155. https://doi.org/10.1186/s40152-014-0014-5
Braje, T.J., Rick, T.C., 2011. Archaeology, historical ecology, and northeastern Pacific pinnipeds and sea otters, in: Braje, T.J., Rick, T.C. (Eds.), *Human Impacts on Seals, Sea Lions, and Sea Otters: Integrating Archaeology and Ecology in the Northeast Pacific*, University of California Press, Oakland, CA, pp. 297–308.

^35^ e.g. Braje, T.J., Rick, T.C., Archaeology, historical ecology, and northeastern Pacific pinnipeds and sea otters. In: Braje, T.J., Rick, T.C. (Eds.), *Human Impacts on Seals, Sea Lions, and Sea Otters: Integrating Archaeology and Ecology in the Northeast Pacific,* University of California Press, Oakland, CA (2011), 297–308.

^36^ e.g. Reitz, E.J., "Fishing down the food web": A case study from St. Augustine, Florida, U.S.A. *American Antiquity* 69 (2004), 63–84. Lenders, H.J.R., Chamuleau, T.P.M., Hendriks, A.J., Lauwerier, R.C.G.M., Leuven, R.S.E.W., Verberk, W.C.E.P., Historical rise of waterpower initiated the collapse of salmon stocks, *Scientific Reports* 6 (2016), 29269. Plank, M.J., Allen, M.S., Nims, R., Ladefoged, T.N., Inferring fishing intensity from contemporary and archaeological size-frequency data, *Journal of Archaeological Science* 93 (2018), 42–53. West, C. F., Etnier, M. A., Barbeaux, S., Partlow, M. A., & Orlov, A. M. (2020). Size distribution of Pacific cod (Gadus macrocephalus) in the North Pacific Ocean over 6 millennia. Quaternary Research, First View. https://doi.org/10.1017/qua.2020.70. Braje, T.J., Rick, T.C., 2011. Archaeology, historical ecology, and northeastern Pacific pinnipeds and sea otters, in: Braje, T.J., Rick, T.C. (Eds.), *Human Impacts on Seals, Sea Lions, and Sea Otters: Integrating Archaeology and Ecology in the Northeast Pacific*, University of California Press, Oakland, CA, pp. 297–308. Reeder-Myers, L., Braje, T.J., Hofman, C.A., Smith, E.A.E., Garland, C.J., Grone, M., Hadden, C.S., Hatch, M., Hunt, T., Kelley, A., LeFebvre, M.J., Lockman, M., McKechnie, I., McNiven, I.J., Newsom, B., Pluckhahn, T., Sanchez, G., Schwadron, M., Smith, K.Y., Smith, T., Spiess, A., Tayac, G., Thompson, V.D., Vollman, T., Weitzel, E.M., Rick, T.C. Indigenous oyster fisheries persisted for millennia and should inform future management. *Nature Communications* 13 (2022), 2383.

^37^ McNeil, J.R. Cheap energy and ecological teleconnections of the industrial revolution, 1780–1920. *Environmental History*, 24 (2019), 492–503

^38^ Barrett, J.H., Locker, A.M., Roberts, C.M., 'Dark Age Economics' revisited: The English fish bone evidence AD 600–1600. *Antiquity* 78 (2004), 618–636. Orton, D. C., Morris, J., Locker, A., & Barrett, J. H., Fish for the city: meta-analysis of archaeological cod remains and the growth of London's northern trade. *Antiquity* 88, nº340 (2014), 516–530. Barrett, J.H., An environmental (pre)history of European fishing: Past and future archaeological contributions to sustainable fisheries. *Journal of Fish Biology* (2019), 1–12.

^39^ Makowiecki, D., D.C. Orton, and J.H. Barrett. Cod and Herring in Medieval Poland. *Cod and Herring*, 117–32.

^40^ Van Neer, Wim, and Anton Ervynck. The Rise of Sea-Fish Consumption in Inland Flanders (Belgium). *Cod and Herring*, 156–71.

^41^ Harland, Jennifer F., A.K.G. Jones, D.C. Orton, and J.H. Barrett. Fishing and Fish Trade in Medieval York: The Zooarchaeological Evidence. *Cod and Herring*, 172–204. Dierickx, K., Presslee, S., Hagan, R., Oueslati, T., Harland, J., Hendy, J., Orton, D., Alexander, M., Harvey, V. Peptide mass fingerprinting of preserved collagen in archaeological fish bones for the identification of flatfish in European waters. *Royal Society Open Science* 9 (2022), 220149.

^42^ Barrett, J.H., A.M. Locker, and C.M. Roberts. ‘Dark Age Economics’ Revisited: The English Fish Bone Evidence Ad 600–1600. *Antiquity* 78 (2004a): 618–36. Barrett, James H. (ed.), *Being an Islander: Production and Identity at Quoygrew, Orkney, Ad 900–1600*. Cambridge: McDonald Institute for Archaeological Research, 2012.

^43^ Star, Bastiaan, Sanne Boessenkool, Agata T. Gondek, Elena A. Nikulina, Anne Karin Hufthammer, Christophe Pampoulie, Halvor Knutsen, et al. Ancient DNA Reveals the Arctic Origin of Viking Age Cod from Haithabu, Germany. *Proceedings of the National Academy of Sciences* 114 (2017): 9152–57.

^44^ Enghoff, Inge Bødker. Fishing in the Baltic Region from the 5th Century Bc to the 16th Century AD: Evidence from Fish Bones. *Archaeofauna* 8 (1999): 41–85. Enghoff, Inge Bødker. Fishing in the Southern North Sea Region from the 1st to the 16th Century AD: Evidence from Fish Bones. *Archaeofauna* 9 (2000): 59–132. Enghoff, Inge Bødker. Herring and Cod in Denmark. *Cod and Herring*, 133–55. Wickler, Stephen, and Lars Narmo. Tracing the Development of Fishing Settlement from the Iron Age to the Modern Period in Northern Norway: A Case Study from Borgvær in the Lofoten Islands. *Journal of Island & Coastal Archaeology* 9 (2014): 72–87. Boethius, Adam, Knut Andreas Bergsvik, and Björn Nilsson. Knowledge from the Ancient Sea – a Long-Term Perspective of Human Impact on Aquatic Life in Mesolithic Scandinavia. *The Holocene* 30 (2020): 632–45.

^45^ *E.g.*, Murphy, Peter. *The English Coast: A History and Prospect*. London: Continuum, 2009. O'Sullivan, Aidan. Place, Memory and Identity among Estuarine Fishing Communities: Interpreting the Archaeology of Early Medieval Fish Weirs. *World Archaeology* 35 (2004): 449–68. Cooper, Lynden P., and Susan Ripper. *Fishing and Managing the Trent in the Medieval Period (7th–14th Century): Excavations at Hemington Quarry (1998–2000), Castle Donington, UK.* Oxford: BAR Publishing, 2017.

^46^ *E.g.*, Gardiner, M., R. Cross, N MacPherson-Grant, and I. Riddler. Continental Trade and Non-Urban Ports in Mid-Anglo-Saxon England: Excavations at Sandton, West Hythe, Kent. *The Archaeological Journal* 158 (2001): 255–61. Dobney, K., D. Jaques, J. Barrett, and C. Johnstone. *Farmers, Monks and Aristocrats: The Environmental Archaeology of an Anglo-Saxon Estate Centre at Flixborough, North Lincolnshire, UK.* Oxford: Oxbow Books, 2007. Barrett, J.H., A.M. Locker, and C.M. Roberts. ‘Dark Age Economics’ Revisited: The English Fish Bone Evidence Ad 600–1600. *Antiquity* 78 (2004a): 618–36. Reynolds, R. The Social Complexities of Early Marine Fish Consumption: New Evidence from Southeast England. *Cod and Herring: The Archaeology and History of Medieval Sea Fishing*, eds. J. Barrett and D. Orton. Oxbow Books, Oxford (2016), 215–20. Makowiecki, D., Orton, D. C., & Barrett, J. H. (2016). Cod and herring in medieval Poland. In J. Barrett & D. Orton (Eds.), Cod and Herring: The Archaeology and History of Medieval Sea Fishing (pp. 117–132). Oxbow Books. Atmore, L. M., Martínez-García, L., Makowiecki, D., André, C., Lõugas, L., Barrett, J. H., & Star, B. (2022). Serial depletion of Baltic herring since the Viking Age revealed by ancient DNA and population genomics. bioRxiv, 2022.05.18.492457. https://doi.org/10.1101/2022.05.18.492457

^47^ Barrett, J.H., A.M. Locker, and C.M. Roberts. The Origin of Intensive Marine Fishing in Medieval Europe: The English Evidence. *Proceedings of the Royal Society B* 271 (2004b): 2417–21.

^48^ Barrett, James H., David Orton, Cluny Johnstone, Jennifer Harland, Wim Van Neer, Anton Ervynck, Callum Roberts, et al. Interpreting the Expansion of Sea Fishing in Medieval Europe Using Stable Isotope Analysis of Archaeological Cod Bones. *Journal of Archaeological Science* 38 (2011): 1516–24.

^49^ Barrett, J.H. Medieval Sea Fishing Ad 500–1550: Chronology, Causes and Consequences. *Cod and Herring*, 250–71.

^50^ Hoffmann, R.C. Economic Development and Aquatic Ecosystems in Medieval Europe. *The American Historical Review* 101, no. 3 (1996): 631–69.

^51^ *E.g.,* Barrett, J.H., A.M. Locker, and C.M. Roberts. 'Dark Age Economics' Revisited: The English Fish Bone Evidence Ad 600–1600. *Antiquity* 78 (2004a): 618–36.

^52^ Harland, Jennifer F., A.K.G. Jones, D.C. Orton, and J.H. Barrett. Fishing and Fish Trade in Medieval York: The Zooarchaeological Evidence. *Cod and Herring*, 172–204.

^53^ *E.g.*, Kowaleski, Maryanne. The Early Documentary Evidence for the Commercialisation of the Sea Fisheries in Medieval Britain. *Cod and Herring*, 23–41.

^54^ Orton, David, James Morris, and Alan Pipe. Catch Per Unit Research Effort: Sampling Intensity, Chronological Uncertainty, and the Onset of Marine Fish Consumption in Historic London. *Open Quaternary* 3 (2017): 1–20.

^55^ Hoffmann, R.C. Economic Development and Aquatic Ecosystems in Medieval Europe. *The American Historical Review* 101, no. 3 (1996): 631–69. Woolgar, C. M. Food and the Middle Ages. *Journal of Medieval History* 36, no. 1 (3// 2010): 1–19. Lenders, H. J. R., T. P. M. Chamuleau, A. J. Hendriks, R. C. G. M. Lauwerier, R. S. E. W. Leuven, and W. C. E. P. Verberk. Historical Rise of Waterpower Initiated the Collapse of Salmon Stocks. *Scientific Reports* 6 (2016): 29269. Gertrud Haidvogl, Historic Milestones of Human River Uses and Ecological Impacts, in: *Riverine Ecosystem Management*, ed. Schmutz S., Sendzimir J. (Aquatic Ecology Series, vol 8) (Springer, Cham. 2018) https://doi.org/10.1007/978-3-319-73250-3_2.

^56^ Woolgar, C. M. Food and the Middle Ages. *Journal of Medieval History* 36, no. 1 (3// 2010): 1–19. Barrett, James H., David Orton, Cluny Johnstone, Jennifer Harland, Wim Van Neer, Anton Ervynck, Callum Roberts *, et al.* Interpreting the Expansion of Sea Fishing in Medieval Europe Using Stable Isotope Analysis of Archaeological Cod Bones. *Journal of Archaeological Science* 38 (2011): 1516–24. Leggett, S. A hierarchical meta-analytical approach to western European dietary transitions in the first millennium AD. *European Journal of Archaeology* http://doi.org/10.1017/eaa.2022.23 (2022): 1–21.

^57^ Jahnke, Carsten. The Medieval Herring Fishery in the Western Baltic. *Beyond the Catch: Fisheries of the North Atlantic, the North Sea and the Baltic, 900–1850*, ed. L. Sicking and D. Abreu-Ferreira, 157–86. Leiden: Brill, 2009. Nedkvitne, Arnved. *The German Hansa and Bergen 1100–1600*. Köln: Böhlau Verlag, 2014. Barrett, J.H. Medieval Sea Fishing Ad 500–1550: Chronology, Causes and Consequences. *Cod and Herring*, 250–71.

^58^ Holm, P. Commercial Sea Fisheries in the Baltic Region C. Ad 1000–1600. *Cod and Herring*, 13–22.

^59^ Campbell, J. Domesday Herrings. *East Anglia's History*, eds. C. Harper-Bill, Carole Rawcliffe and Richard G. Wilson, 5–17. Woodbridge: Boydell, 2002. Kowaleski, Maryanne. The Early Documentary Evidence for the Commercialisation of the Sea Fisheries in Medieval Britain. *Cod and Herring*, 23–41.

^60^ Barrett, J.H., Locker, A.M., Roberts, C.M., 'Dark Age Economics' revisited: The English fish bone evidence AD 600–1600. *Antiquity* 78 (2004), 618–636. *Cod and Herring*, 250–271.

^61^ T. Bekker-Nielsen (2016), Ancient Fishing and Fish Processing in the Black Sea Region. *The Inland Seas. Towards an Ecohistory of the Mediterranean and the Black Sea*, eds. T. Bekker-Nielsen & R. Gertwagen. *Geographica Historica* 35 (Stuttgart: Franz Steiner Verlag) 287–308.

^62^ Jazwa, C.S., Joslin, T.L., Kennett, D.J., Fishing, subsistence change, and foraging strategies on western Santa Rosa Island, California. *American Antiquity* 85 (2020), 591–608.

^63^ Friesen, M., Pan-Arctic population movements: The early Paleo-Inuit and Thule Inuit migrations. In: Friesen, M., Mason, O. (Eds.), *The Oxford Handbook of the Prehistoric Arctic*, Oxford University Press, Oxford (2016), 673–691.

^64^ Sear, D.A., Allen, M.S., Hassall, J.D., Maloney, A.E., Langdon, P.G., Morrison, A.E., Henderson, A.C.G., Mackay, H., Croudace, I.W., Clarke, C., Sachs, J.P., Macdonald, G., Chiverrell, R.C., Leng, M.J., Cisneros-Dozal, L.M., Fonville, T., Pearson, E., Human settlement of East Polynesia earlier, incremental, and coincident with prolonged South Pacific drought, *PNAS* 117 (2020), 8813–8819.

^65^ Marzano, A. (2013), *Harvesting the Sea. The Exploitation of Marine Resources in the Roman Mediterranean* (Oxford: Oxford University Press). Bekker-Nielsen, T. & Gertwagen, R. (eds.), *The Inland Seas: Towards an Ecohistory of the Mediterranean and the Black Sea*. (Geographica Historica 35). Stuttgart: Franz Steiner Verlag (2016).

^66^ Holm, P., F. Ludlow, C. Scherer, C. Travis, B. Allaire, C. Brito, P. W. Hayes, J. A. Matthews, K. Rankin, R. Breen, R. Legg, K. Lougheed & Nicholls. J., The North Atlantic Fish Revolution (ca AD 1500). *Quaternary Research* (2019) https://doi.org/10.1017/qua.2018.153: 15. Holm, P., Nicholls, J., Hayes, P. W, Ivinson, J & Allaire, B. (2022), Accelerated Extractions of North Atlantic Cod and Herring 1520–1790. *Fish and Fisheries* 23:1, 54–72. DOI: 10.1111/faf.12598

^67^ Richards, R., New market evidence on the depletion of southern fur seals: 1788–1833. *New Zealand Journal of Zoology* 30, nº1 (2003), 1–9.

^68^ Jones, T.L., Culleton, B.J., Larson, S., Mellinger, S., Porcasi, J.F., Toward a Prehistory of the Southern Sea Otter (Enhydra lutris nereis), in: Braje, T.J., Rick, T.C. (Eds.) *Human Impacts on Seals, Sea Lions, and Sea Otters: Integrating Archaeology and Ecology in the Northeast Pacific*, University of California Press, Oakland, CA (2011), 243–271. Estes, J.A., Burdin, A., Doak, D.F., Sea otters, kelp forests, and the extinction of Steller’s sea cow, *PNAS* 113 (2016), 880–885.

^69^ Barkham, S.H. (1984) *Op. Cit.*. Barkham, M.H., La industria pesquera en el País Vasco peninsular al principio de la Edad Moderna: ¿una edad de oro? *ITSAS Memoria. Revista de Estudios Marítimos del País Vasco* 3 (2000), 29–75. Brito, C., Vieira, N., Jordão, V. & Teixeira, A., Digging into our whaling past: Addressing the Portuguese influence in the early modern exploitation of whales in the Atlantic. In *Environmental History in the Making*. Bern: Springer International (2016), 33–47.

^70^ Nordin, J., The centre of the world: The material construction of Eurocentric domination and hybridity in a Scandinavian 17th-century context. *Journal of Material Culture*, 18, nº2 (2013), 189–209.

^71^ Vilar, P., *A History of Gold and Money, 1450–1920*. London: Verso (1991). Elliott, J.H., *Empires of the Atlantic World: Britain and Spain in America 1492–1830*, Yale U.P. (2006). Drelichman, M. & Voth, H.-J., The Sustainable Debts of Philip II: A Reconstruction of Spain's Fiscal Position, 1560–1598. *Centre for Economic Policy Research, Discussion Paper* (DP6611) (2007). Findlay, R. & O’Rourke, K. H., *Power and Plenty: Trade, War, and the World Economy in the Second Millennium*. Princeton: Princeton University Press (2007), 143–226.

^72^ Quinn, D.B., *Explorers and colonies: America, 1500–1625*. London (Hambledon Press) (1990), 301.

^73^ Pope, P. (2004). *Fish into wine: The Newfoundland plantation in the seventeenth century.* Chapel Hill, University of North Carolina Press.

^74^ Holm et al. (2022).

^75^ P. Holm, Atlantic Wealth. Keynote address, Congress of Nordic Historians, August 2022 (unpublished).

^76^ The need to revise models based on the new landings data is argued by P. Holm, P. W. Hayes & J. Nicholls (2022), Comment on “Five centuries of cod catches in Eastern Canada”, by Schijns *et al.*, *ICES Journal of Marine Science*, https://doi:10.1093/icesjms/fsac089. See also the response by R. Schijns, R. Froese, J. Hutchings & D. Pauly (2022) Reply to Holm *et al.* 2022, “Comment on ‘Five centuries of cod catches in eastern Canada,’ by Schijns *et al.*” *ICES Journal of Marine Science* https://doi.org/10.1093/icesjms/fsac090

^77^ Nicholls, J., Allaire, B. & Holm, P., The Capacity Trend Method: A new approach for enumerating the Newfoundland cod fisheries (1675–1790). *Historical Methods: A Journal of Quantitative and Interdisciplinary History* (2021), DOI: 10.1080/01615440.2020.1853643.

^78^ *Cod and Herring*. Barrett, J.H., Boessenkool, S., Kneale, C.J., O’Connell, T.C., Star, B., Ecological globalisation, serial depletion and the medieval trade of walrus rostra, *Quaternary Science Reviews* 229 (2020), 106122.

^79^ Jones, R., Sea otters and savages in the Russian Empire: The Billings Expedition, 1785–1793. *Journal for Maritime Research* 8, nº1 (2006), 106–121. Braje, T.J., Rick, T.C., 2011. Archaeology, historical ecology, and northeastern Pacific pinnipeds and sea otters, in: Braje, T.J., Rick, T.C. (Eds.), Human Impacts on Seals, Sea Lions, and Sea Otters: Integrating Archaeology and Ecology in the Northeast Pacific, University of California Press, Oakland, CA, pp. 297–308.

^80^ Barkham, M.H., El comercio marítimo viscaíno y guipuzcoana con el Atlántico Peninsular (Asturias, Galicia, Portugal Y Andaluzia) y con los archipiélagos de Canarias y Madeira, al principio da le Edad Moderna. *ITSAS Memoria. Revista de Estudios Marítimos del País Vasco* 4 (2003), 147–164. Szabo, V., *Monstrous Fishes and the Mead-Dark Sea. Whaling in the Medieval North Atlantic*. Leiden, Boston: Brill (2008). Rodrigues, A., Charpentier, A., Bernal-Casasola, D., Gardeisen, A., Nores, C., Antonio, J., Millán, P., Mcgrath, K. & Speller, C., Forgotten Mediterranean calving grounds of grey and North Atlantic right whales: evidence from Roman archaeological records. *Proc. R. Soc. B* 285 (2018), 20180961. Brito, C., Beauties and Beasts: Whales in Portugal, from Early-Modern Monsters to Today’s Flagship Species. Environment & Society Portal, *Arcadia*, no. 21. Rachel Carson Center for Environment and Society (2018) ISSN 2199-3408.

^81^ York, R. (2017), Why Petroleum Did Not Save the Whales. *Socius* 3:1–13. Brunner, B. *The Ocean at Home: An Illustrated History of the Aquarium*. Princeton Architectural Press (2005).

^82^ Flores, J., The Iberian Empires, 1400 to 1800. In Jerry H. Bentley, Sanjay Subrahmanyam & Merry E. Wiesner-Hanks (eds). *The Cambridge World History*. Volume VI. The Construction of a Global World, 1400–1800 CE. Part I: Foundations. Cambridge University Press (2015), 271–296. Marks, R.B., "Exhausting the Earth": Environment and History in the Early Modern World. In Jerry H. Bentley, Sanjay Subrahmanyam & Merry E. Wiesner-Hanks (eds). *The Cambridge World History*. Volume VI. The Construction of a Global World, 1400–1800 CE. Part I: Foundations. Cambridge University Press (2015), 29–53.

^83^ Teich, M., Porter, R. & Gustafsson, B. (eds), *Nature and Society in Historical Context*. Cambridge University Press (1997). MacGregor, A. (ed), *Naturalists in the field: Collecting, recording and preserving the natural world from the Fifteenth to the Twenty-First Century*. Leiden, Boston: Brill (2018).

^84^ Williams, M.J., Ausubel. J., Poiner I., Garcia S.M., Baker D.J., Clark M.R., *et al.*, Making Marine Life Count: A New Baseline for Policy. *PLoS Biol* 8, nº10 (2010), e1000531.

^85^ Springer, A., Estes, J., van Liet. G., Williams, T., Doak, D., Danner, E., Forney, K. & Pfister, B., Sequential megafaunal collapse in the North Pacific Ocean: an ongoing legacy of industrial whaling? *PNAS* 100, nº 21 (2003), 12223–12228. DeMaster D., Trites A., Clapham P., MizrochS, Wade P., Small R & Hoef J., The sequential megafaunal collapse hypothesis: testing with existing data. *Progress in Oceanography* 68, nº2–4 (2006), 329–342.

^86^ Freytag, N., Nature and Environment in Early Modern Europe. Brewminate. Posted January, 5, 2019. https://brewminate.com/nature-and-environment-in-early-modern-europe/. Vieira, N. *A taxonomia da baleação portuguesa entre os séculos XV e XVIII: Uma história atlântica do mar, das baleias e das pessoas*. Faculdade de Ciências Sociais e Humanas da Universidade NOVA de Lisboa, 435 p (2020 *)*.

^87^ Warsh, M. A. *American baroque: pearls and the nature of empire, 1492–1700*. Chapel Hill: The University of North Carolina Press (2018).

^88^ Barrera-Osorio, A. & Olarte, M.N., Ciencia, tecnología, saberes locales e imperio en el mundo atlántico, siglos XV-XIX. *Historia Crítica* 73 (2019), 3–20.

^89^ Sánchez Martínez, A. Artesanos, cartografía e imperio. La producción social de un instrumento náutico en el mundo ibérico, 1500–1650. Historia Crítica, (2019), 21–41, (73).

^90^ Cañizares-Esguerra, J. *Católicos y puritanos en la colonización de América*, Madrid, Marcial Pons, (2008). León, P.S. *Abundacia y frustración: Por una historia conceptual de la economia en la modernidade latino-americana*. Madrid, Postmetropolis. Colección Metro, 3 (2015). Vieira, N. *A Comparative Approach to Historical Whaling Techniques: Transfer of Knowledge in the 17th century from the Biscay to Brazil*. In A Polónia, F Bracht, GC Conceição & M Palma (eds.). Cross-cultural Exchange and the Circulation of Knowledge in the First Global Age. 1st ed. Porto, CITCEM/Edições Afrontamento (2018), 125–143. Torres, P.U. Paisajes del confín territorial. Las Relaciones Geográficas del siglo XVI como fuentes para la historia ambiental. *HALAC, Historia Ambiental, Latinoamericana y Caribeña*, 11, n. 1 (2021), 130–155. Warsh, M. A. *American baroque* (2018).

^91^ Barrera-Osorio, A., *Experiencing Nature. The Spanish American Empire and the Early Scientific Revolution*. Austin: University of Texas Press (2006). Alves, A.A., *The animals of Spain: An introduction to imperial perceptions and human interaction with other animals, 1492–1826*. Leiden, Boston: Brill (2011). Cañizares-Esguerra, J. & Breen, B., Hybrid Atlantics: Future Directions for the History of the Atlantic World. *History Compass* 11, nº8 (2013), 597–609. Buschmann, R. F., *Iberian Visions of the Pacific Ocean, 1507–1899*. Basingstoke: Palgrave Macmillan (2014).

^92^ Romero, A. & Agudo, A.I., The scientific discovery of the Amazon river dolphin *Inia geoffrensis*. *Marine Mammal Science* 13, nº3 (1997), 419–426. Herold, B., Horst, T. & Leitão, H., The “Natural History of Portugal” by Leonhard Thurneysser zum Thurn, ca. 1555–1556. *Ágora. Estudos Clássicos em Debate*, 19 (2017), 305–334.

^93^ Delliaux M. & Gautier A., Cheval ou baleine? Les noms du morse dans les mondes septentrionaux (IX ^e^-milieu du XVI ^e^ siècle), in Jacquemard C., Gauvin B., Lucas-Avenel M.-A., Clavel B. & Buquet T. (éds), Animaux aquatiques et monstres des mers septentrionales (imaginer, connaître, exploiter, de l’Antiquité à 1600). *Anthropozoologica* 53 (15), (2018), 175–183.

^94^ Casset M., La consommation de produits de la mer à la cour du duc de Bretagne pendant son exil en Angleterre (1377–1378), in Jacquemard C., Gauvin B., Lucas-Avenel M.-A., Clavel B. & Buquet T. (éds), Animaux aquatiques et monstres des mers septentrionales (imaginer, connaître, exploiter, de l’Antiquité à 1600). *Anthropozoologica* 53 (5), (2018), 73–80.

^95^ Carney, J., “The mangrove preserves life”: Habitat of African survival in the Atlantic world. *Geographical Review* 107, nº3 (2017), 433–451. Warsh, M.A. (2018) *Op. Cit.*. Vieira, N. & Brito, C. (2017) *Op. Cit..*

^96^ Vieira, N., *A taxonomia da baleação portuguesa entre os séculos XV e XVIII: Uma história atlântica do mar, das baleias e das pessoas*. Faculdade de Ciências Sociais e Humanas da Universidade NOVA de Lisboa (2020).

^97^ Catterall, D. & Campbell, J. (eds), *Women in Port: Gendering Communities, Economies, and Social Networks in Atlantic Port Cities, 1500–1800.* Leiden, Boston: Brill (2012).

^98^ Brito, C., People, Manatees and the Aquatic Environment in Early Modern Americas: Confluence and Divergence in the Historical Relationships Between Humans and Animals. *Revista Brasileira de História* (2019), 1–21.

^99^ Vasconcellos, S., *Noticias curiosas, e necessarias das cousas do Brasil*. Livro I. Lisboa: Na officina de Ioam de Costa (1668). Durand, J., *Ocaso de Sirenas*: Manaties en el siglo XVI. México: Tezontle (1950). Veríssimo, J., *A Pesca na Amazónia*. Universidade Federal do Pará (1970). Vieira, N. & Brito, C. (2017) *Op. Cit..*

^100^ For West Africa, references to ‘ *pesce-dona*’, ‘ *peixe-mulher*’ or woman-fish, the Kikongo *Ngulu-a-Maza*, are found in the work by the Italian friar Giovanni Antonio Cavazzi: Cavazzi, G.A. (1621–1678 *). Istorica descrizione de' tre' regni Congo, Matamba, et Angola*. Bologna: Giacomo Monti (1687).

^101^ Brito, C., Connected margins and disconnected knowledge: Exotic marine mammals in the making of early modern European natural history. In Amélia Polónia; Fabiano Bracht; Gisele C. Conceição; Monique Palma (eds). *Cross-cultural Exchange and the Circulation of Knowledge in the First Global Age*. Porto: CITCEM/Edições Afrontamento, v.1 (2018), 106–132. See also Canadelli, E., Storia di una collezione settecentesca. Il Museo vallisneriano dell’Università di Padova attraverso i cataloghi storici. *Museologia Scientifica Memoria*, 17 (2017), 34–38. Egmond, F. *Eye for detail: Images of plants and animals in art and science 1500–1630*. Reaktion Books (2017). An important window to early modern perception of natural history Adriaen Coenen’s Fish Book (1580) [ https://galerij.kb.nl/kb.html#/en/visboek/page/15/zoom/3/lat/-55.17886766328199/lng/52.55859375] and *The Whale Book: Whales and other marine animals as described by Adriaen Coenen in 1585*. With an introduction, translation and comments by Florike Egmond and Peter Mason. London: Reaktion Book (2003) [1585].

^102^ This literature (from environmental anthropologists and others) is now voluminous, but see Sillitoe, P. (ed), *Indigenous Knowledge: Enhancing its Contribution to Natural Resources Management*. Cambridge & Wallingford: CABI Publishing (2017).

^103^ Francis, R.C., Historical approaches to the Northern California Current Ecosystem. Holm, P, *et al*., (eds.). *The Exploited Seas: New Directions for Marine Environmental History.* Research in Maritime History. St. John's, Newfoundland (2001), 123–140.

^104^ Holm, P. *et al.*, The North Atlantic Fish Revolution (ca AD 1500).

^105^ Utterström, G., Migratory Labour and the Herring Fisheries of Western Sweden in the 18th century. *Scandinavian Economic History Review* 7, nº1 (1959), 3–40.

^106^ Nilsson, L., Det stora sillfisket 1752–1808. E. Lönnroth (ed.), *Bohusläns historia, Göteborg* (1963) *.* Holm, P., The Bohuslen Herring. Interlude to Dutch supremacy in the European fish market. L. M. Akveld (ed.). In: *In het kielzog*. Amsterdam: De Bataafsche Leeuw (2003), 282–288. Enflo, K. & Missiaia, A., Regional GDP estimates for Sweden, 1571–1850. *Historical Methods*, 51, nº2 (2018), 115–137.

^107^ Sahrhage, D. & Lundbeck, J., *A history of fishing*. Berlin: Springer-Verlag (1992), 72.

^108^ Innis, H., Rise and fall of the Spanish fishery in Newfoundland. *Proc. & Trans. Royal Soc. of Canada* 3, nº25 (1931), 51–70. Auty, R.M., *Sustaining Development in Mineral Economies: The Resource Curse Thesis*. London: Routledge (1993). Frankel, J.A., *The Natural Resource Curse: A Survey.* National Bureau of Economic Research Working Paper (2010), 15836.

^109^ Alheit, J. & Hagen, E., Long-term climate forcing of European herring and sardine populations. *Fisheries Oceanography,* 6 (1997), 130–139. Corten, A., A proposed mechanism for the Bohuslan herring periods. *ICES Journal of Marine Science* 56 (1999), 207–220.

^110^ Schurer, A. P., Hegerl, G. C., Luterbacher, J., Brönnimann, S., Cowan, T., Tett, S. F. B., Zanchettin, D. & Timmreck, C. (2019), Disentangling the causes of the 1816 European year without a summer. *Environmental Research Letters* 14, 094019.

^111^ Alexander, K. E., Leavenworth, W. B., Willis, T. V., Hall, C., Mattocks, S., Bittner,1., Klein, E., Bryan, A., Rosset,J., Carr B. H. & Jordaan Z. (2017), Tambora and the mackerel year: Phenology and fisheries during an extreme climate event. *Science Advances* 3, e1601635

^112^ Sigl, M., Winstrup, M., McConnell, J.R., Welten, K.C., Plunkett, G., Ludlow, F., Büntgen, U., Caffee, M., Chellman, N., Dahl-Jensen, D., Fischer, H., Kipfstuhl, S., Kostick, C., Maselli, O.J., Mekhaldi, F., Mulvaney, R., Muscheler, R., Pasteri, D.R., Pilcher, J.R., Salzer, M., Schüpbach, S., Steffensen, J.P., Vinther, B., Woodruff, T.E. (2015), Timing and Climate Forcing of Volcanic Eruptions during the Past 2,500 years, *Nature*, 523, 543–549.

^113^ J. Slawinska & A. Robock (2018) Impact of volcanic eruptions on decadal to centennial fluctuations of Arctic sea ice extent during the last millennium and on initiation of the Little Ice Age. *Journal of Climate* 31, 2145–2167.

^114^ For examples, see: Lin, I.‐I.. Hu, C., Li, Y.-H., Ho, T.-Y., Fischer, T. P., Wong, G. T. F., Wu, J., Huang, C.-W., Chu, D. A., Ko, D. S. & Chen, J.-P. (2011) Fertilization potential of volcanic dust in the low‐nutrient low‐chlorophyll western North Pacific subtropical gyre: Satellite evidence and laboratory study, *Global Biogeochemical Cycles*, 25, GB1006. Lindenthal, A., Langmann, B., Pätsch, J., Lorkowski, I., & Hort, M. (2013) The ocean response to volcanic iron fertilisation after the eruption of Kasatochi volcano: A regional-scale biogeochemical ocean model study, *Biogeosciences*, 10(6), 3715–3729. While the North Sea is certainly not as nutrient limited as many larger oceanic regions more remote from land and associated nutrient run-off, considerable periodic volcanic fallout from nearby Iceland is worth consideration as a contributor to the Bohuslän phenomenon. For Icelandic volcanic history in recent centuries and millennia, see T. Thordarson and G. Larsen (2007), Volcanism in Iceland in historical time: Volcano types, eruption styles and eruptive history, *Journal of Geodynamics*, 43, 118–52.

^115^ Ludlow, F., Stine, A. R., Leahy, P., Murphy, E., Mayewski, P., Taylor, D., Killen, J., Baillie, M., Hennessy, M. and Kiely, G. (2013). Medieval Irish Chronicles Reveal Persistent Volcanic Forcing of Severe Winter Cold Events, 431–1649 CE, *Environmental Research Letters*, 8 (2), L024035. Pausata, F. S. R., Chafik, L., Caballero, R. & Battisti, D. S (2015). Impacts of a high-latitude volcanic eruption on ENSO and AMOC. *Proceedings of the National Academy of Sciences,* 112(45), 13784–13788. This effect can depend upon variables such as the location of the volcano in question, with summer cooling but winter air-temperature warming over northern hemispheric land masses observed after some tropical eruptions, shifting to a net cooling in the second and subsequent winters.

^116^ Beaugrand, G. & Reid, P. (2003). Long-term changes in phytoplankton, zooplankton and salmon related to climate. *Global Change Biology* 9(6), 801–817.

^117^ Dudden, A., The Sea of Japan/Korea’s East Sea, in Armitage, D., Bashford, A & Sivasundaram, S. (eds), *Oceanic Histories*. Cambridge: Cambridge University Press (2017), 182–208, 186.

^118^ Atmore, L. M., Aiken, M., & Furni, F. (2021). Shifting baselines to thresholds: Reframing exploitation in the marine environment. Frontiers in Marine Science, 8, 742188. https://doi.org/10.3389/fmars.2021.742188

^119^ Betts, M., Zooarchaeology and the reconstruction of ancient human-animal relationships in the Arctic. In M. Friesen & O. Mason (eds.), *The Oxford Handbook of the Prehistoric Arctic*. Oxford: Oxford University Press (2016).

^120^ Broeze, F., *Island Nation: A History of Australians and the Sea.* St. Leonards: Allen & Unwin (1998).

^121^ Acebes, J.M.V., *Hunting “Big Fish”: a marine environmental history of a contested fishery in the Bohol Sea*. PhD thesis, Murdoch University (2013).

^122^ Santana Perez, J.M., Pesca y Ciudades Canarias en el Antiguo Régimen. *Coloquios de Historia Canario Americana* (2010), 1051–1062.

^123^ Richards, R., New market evidence on the depletion of southern fur seals: 1788–1833. *New Zealand Journal of Zoology* 30, nº1 (2003), 1–9.

^124^ Smith, D., *Oyster. A World History.* London: The History Press (2010).

^125^ Ellis, M., *A baleia no Brasil colonial. Feitorias, baleeiros, técnicas, Monopólio, Comércio, Iluminação*. São Paulo: Melhoramentos (1969). Davis, L.E. *et al.*, *In Pursuit of Leviathan: technology, institutions, productivity, and profits in American whaling, 1816–1906.* Chicago: University of Chicago Press (1997). Hart, I. & Edmundson, W., *A History of Whaling in Brazil. From royal fish to Japanese delicacy*. Pequena, Herefordshire (2017). Vieira, N. (2020) *Op. Cit.*. Laist, D.W., *North Atlantic Right Whales: From hunted Leviathan to conservation icon*. Baltimore: Johns Hopkins University Press (2017).

^126^ Robards, M.D. & Reeves, R.R., The global extent and character of marine mammal consumption by humans: 1970–2009. *Biological Conservation* 144, nº12 (2011), 2770–2786.

^127^ Preliminary production data are published in Nicholls, J., Allaire, B. & Holm, P., The Capacity Trend Method: A new approach for enumerating the Newfoundland cod fisheries (1675–1790). Historical Methods: A Journal of Quantitative and Interdisciplinary History (forthcoming).

^128^ e.g. Orton, D., Morris, J., Pipe, A., Catch per unit research effort: sampling intensity, chronological uncertainty, and the onset of marine fish consumption in historic London. *Open Quaternary* 3 (2017), 1–20.

^129^ e.g. Bleasdale, M., Ponce, P., Radini, A., Wilson, A.S., Doherty, S., Daley, P., Brown, C., Spindler, L., Sibun, L., Speller, C., Alexander, M.M., Multidisciplinary investigations of the diets of two post-medieval populations from London using stable isotopes and microdebris analysis. *Archaeological and Anthropological Sciences* 11 (2019), 6161–6181.

^130^ Josephson, E., Smith, T. & Reeves, R., Historical distribution of right whales in the North Pacific. *Fish and Fisheries* 9, nº2 (2008), 155–168. Richards, R., Past and present distributions of southern right whales (Eubalaena australis). *New Zealand Journal of Zoology*, 36 (4) (2009), 447–459. Jackson, J.A., Carroll, E.L., Smith, T.D., Zerbini, A.N., Patenaude, N.J. & Baker, C.S., An integrated approach to historical population assessment of the great whales: Case of the New Zealand Southern right whale. *Royal Society Open Science*, 3, nº3 (2016), 1–16. Monsarrat, S., Pennino, M.G., Smith, T.D., Reeves, R.R., Meynard, C.N., Kaplan, D.M. & Rodrigues, A.S.L., A spatially explicit estimate of the pre whaling abundance of the endangered North Atlantic right whale. *Conservation biology: the journal of the Society for Conservation Biology* 30, nº4 (2016): 783–791.

^131^ Loewen, B., Historical Data on the Impact of 16th -Century Basque Whaling on Right and Bowhead Whales in the Western North Atlantic. *Canadian Zooarchaeology* 26 (2009), 3–24. Vieira, N. (2020) *Op. Cit.*.

^132^ Tønnessen, J.N & Johnsen, A.O., *The History of Modern Whaling*. University of California Press, Berkeley and Los Angeles (1982). Carvalho, I., Brito, C., dos Santos, M.e. & Rosenbaum, H., The waters of São Tomé: a calving ground for West African humpback whales? *African Journal of Marine Science* 33, nº1 (2011), 91–97.

^133^ Rodrigues ASL, Charpentier A, Bernal-Casasola D, Gardeisen A, Nores C, PisMilla ´n JA, McGrath K, Speller C.F,. Forgotten Mediterranean calving grounds of grey and North Atlantic right whales: evidence from Roman archaeological records. *Proc. R. Soc.* B285 (2018), 20180961.

^134^ For case studies see e.g. B. Allaire & P. Holm (2022), The price of fish in French supply contracts (1538–11751). A quantitative approach to the early modern French fish market. Food & History 20:1 (2022) 41–66. T. Bestor 2004), Tsukiji: The Fish Market at the Center of the World (University of California Press). For the wider significance of fish markets see S. Iudicello (1999) Fish, markets and fishermen : the economics of overfishing (London: Earthscan).

^135^ Orton *et al.* (2014) op. cit. R. Scola (1992), Feeding the Victorian City. The food supply of Manchester, 1770–1870 (Manchester University Press). M. Fabinyi (2011), Historical, cultural and social perspectives on luxury seafood consumption in China. Environmental Conservation 39:1, 83–92.

^136^ Miller, P.N., *The Sea: Thalassography and Historiography.* Ann Arbor: University of Michigan Press (2014). Mukherjee, R., Escape from Terracentrism: Writing a Water History. *Indian Historical Review* 41, nº 11(2014), 87–101. Starkey, D. and M. Hahn-Pedersen (Eds), *Concentration and Dependency:The Role of Maritime Activities in North Sea Communities, 1299–1999.* Esbjerg: Fiskeri- og Søfartsmuseets Studieserie (2002).

^137^ Travis, C., F. Ludlow, A. Matthews, K. Lougheed, K. J. Rankin, B. Allaire, R. J. Legg, P. Hayes, J. Nicholls, L. Towns & Holm, P., Inventing the Grand Banks: A deep chart. Humanities GIS, Cartesian, and literary perceptions of the north‐west Atlantic fishery ca 1500–1800. *Geo: Geography and Environment* (2020), e85.

^138^ See for example O. Hasslöf (1949), *Svenska västkustfiskarna* (Stockholm: Esselte). S. Cadigan, The Moral Economy of the Commons: Ecology and Equity in the Newfoundland Cod Fishery, 1815–1855. *Labour / Le Travail* 43 (1999), 9–42. Holm, P., Historical fishing communities. In: K. Schwerdtner Máñez and B. Poulsen (Eds.), *Perspectives on Oceans Past: A Handbook of Marine Environmental History*. Springer (2016), 31–46.

^139^ Innis, H.A., *The Fur Trade in Canada: An Introduction to Canadian Economic History.* New Haven (1930). Auty, R.M., *Sustaining Development in Mineral Economies: The Resource Curse Thesis* London: Routledge (1993). Frankel, J.A., *The Natural Resource Curse: A Survey*. National Bureau of Economic Research Working Paper 15836 (2010).

^140^ Rankin, K. J. and P. Holm (2019). Cartographical Perspectives on the Evolution of Fisheries in Newfoundland’s Grand Banks Area and Adjacent North Atlantic Waters in the Sixteenth and Seventeenth Centuries. *Terrae Incognitae* 51(3): 190–218.

^141^ Townsend, C.H., The distribution of certain whales as shown by logbook records of American whaleships. 19, *Zoologica* (1935), 1–50. Alden, D., Yankee Sperm Whalers in Brazilian Waters, and the Decline of the Portuguese Whale Fishery (1773–1801). *The Americas* 20, nº 3 (1964), 267–288. Vieira, N., Hayes, P. & Matthews, A., Facing changes, changing targets: Sperm Whale hunting in the 18 ^th^ century Brazil. Environment & Society Portal, *Arcadia* (2019), 44 *.* Rachel Carson Center for Environment and Society.

