# Supplementary material for: New challenges for the Human Oceans Past agenda
Source: Open Res Eur. 2022 Sep 29;2:114. [Version 1] doi: 10.12688/openreseurope.15095.1 (PMC10446148; doi:10.12688/openreseurope.15095.1)
Supplement: Peer review history

# Peer review: Reviewer response for version 1

**DOI:** 10.21956/openreseurope.16323.r30179

**Published:** 2022-10-17

**Authors:** Antonio Ortega Santos

**Affiliations:** 1 University of Granada, Granada, Spain

Reading this magnificent text, one understands even more the urgency of a global research agenda on oceans and seas as an answer to the questions that assail us from the urgency of the environmental and climate crisis. It is a text of a high level of reflection, which allows us to understand the paths travelled for marine research, indicates the points to be completed in the future and provides us with reflections for the future. Scientific soundness, interdisciplinary dialogues and a systematisation of the work towards the future run through the pages of the article with enormous validity. Therefore, with this quality of research, my comments only make sense as proposals for dialogue and collaboration with the authors. They are only reflections born of the interesting elements that are proposed in the text, answers that I also seek in my own research. 

 Environmental historians, with a strong focus on land, agrarian and peasant historians, have much to learn from the challenges presented in this magnificent article.

**Methodological Challenges.**

 In this first section I will express ideas and proposals arising from the methodological profiles proposed in the text. 

*Supporting a Non-Human-Centric Matrix* *.*

 The first of these stems from interdisciplinarity, which is present in the text in all its aspects, but which may have more possible tools. From the field of environmental history in which some of us move, the links with archaeology and history have been and are constants in our daily work. This dialogue is very much embedded in the narrative of the text but it would be good to reinforce the dialogues from Environmental History, the processes of historical co-evolution as opposed to social and environmental determinisms, making the historical processes from the Modern Age to the present more complex. To this end, the text invites us to continue with the History of Animals, of Non-Human Beings in our agenda, which many of us should implement in our research.

*Contributions to a Non-Eurocentric Matrix and Communality*

 A second challenge is the Eurocentric rupture of the text, a logical consequence of the projects that contribute to it. There are many research experiences that we have developed in many other oceans that are not coeval with the logics described in the text, nor do they have the same timelines (in our work in the Pacific Ocean, Baja California Sur, the transition from hunter-gatherers to ocean commodification occurred between 1700 and 1830), nor similar logics of appropriation of goods. We know about this proposal of the authors and it is described in a few paragraphs, but to understand even more about the Oceans we need to look very broadly at the Global South. We are developing projects on shark consumption in Reunion Island, or the difficulty around loggerhead turtle consumption or totoaba in Mexico are just some of the tasks we are engaged in in many places that can bring knowledge to this agenda.

 Linked to this debate, the reflection on the Global Commons applied to marine and oceanic resources, pushes us to reflect on the forms of communality that have survived throughout history, parallel to the implementation of a "capitalisation of Nature" since the mid-18th century. This could be a very fertile field for pooling research on a global scale.

 This openness to research in non-European territories, which in many cases is carried out by researchers from European institutions in addition to our projects in Europe, could allow us to design a greater and better response to global challenges in socio-environmental research on the oceans, given the interaction between many of the processes of change in the management of natural assets.

 How can we create such common global agendas?

*Contributions to a Quantitative Research Matrix*

 In addition to the above, the authors point out very well the limitations to go beyond the Baselines before 1950-60, following FAO statistics. It is true of the difficulty but we can collaborate between different disciplines to establish local projects in the oceans in order to - using the methodology of Material Energy Flow Accounts, MEFA - design local baselines with the use of historical material from archives and a central element, the memory of the inhabitants of the oceans, of the seas... the Biocultural Memory that allows the recovery of traditional knowledge and know-how of the coastal peoples.

 This assessment of territorial sustainability with a historical matrix can resolve this baseline limitation and provide valuable information with which to design a new communality as a social tool for the sustainability of the oceans and seas, with local knowledge. 

 This can facilitate a methodological leap already mentioned, how to investigate, what archival materials we use, how we put into dialogue with tools of participatory and collaborative research, new research agendas in which archaeology, history and anthropology can give other possible results. This proposal may require "decolonising research processes", especially outside European contexts. 

 Can we design platforms of biophysical, social and emotional indicators, from interdisciplinary contributions, for the design of future sustainability tools and provide input to EU public policies?

**Proposals for new dialogues.**

 This magnificent text provides insights to new fields of research in the future, which emerge from the many already indicated in it and from some that are pointed out in it, and we can cite some ideas for the future.

*Production*

 Oceans are (fluid) territories with intense levels of disputes, of environmental conflicts arising from the well-described process of commodification of natural resources (Anthropocene vs. Capitalocene?). This view of the oceans as resource providers, subject to intense extraction processes, should not make us forget that they are avenues for the implementation of new forms of commodification in the Global South: Guano, salt, raw materials, mother-of-pearl and pearls, turtles, underwater mining... are just some of the examples of this old-new intensive exploitation of the seas as such and as "highways for great acceleration". 

*Consumption*

 If we can build historical baselines, pre 60's, it will be easy to establish laboratories in many ocean territories on changes in agri-food systems, changes in diets, changes in human consumption patterns on marine goods. This change in food systems must be connected to changes in agrarian patterns (working with MEFA tools may be easier to assess quantitatively and qualitatively) since for many peoples the agrarian and the marine are integral to consumption patterns.

 Inequalities in consumption levels, the need for a new form of relationship and access to marine goods in the face of ecocide may allow for a new Political Ecology of the Oceans to be generated in which to integrate both social and environmental inequity and the socio-environmental tensions that occur around material hexaction.

 Finally, congratulations to the text for the work, which is enormous and of the highest quality. But above all, congratulations for inviting us to think about our research proposals and to incorporate many of these proposals into projects. I am sure that many dialogues will arise in the future to share.

Is the argument persuasive and supported by appropriate evidence?

Yes

Is the work clearly and cogently presented?

Yes

Is the topic of the essay discussed accurately in the context of the current literature?

Yes

Does the essay contribute to the cultural, historical, social understanding of the field?

Yes

Reviewer Expertise:

Environmental History, Environmental Conflicts, Ocean History

I confirm that I have read this submission and believe that I have an appropriate level of expertise to confirm that it is of an acceptable scientific standard.

# Peer review: Reviewer response for version 1

**DOI:** 10.21956/openreseurope.16323.r30181

**Published:** 2022-10-10

**Authors:** Jack Bouchard

**Affiliations:** 1 History Department, Rutgers University, New Brunswick, NJ, USA

This is a welcome, well-conceived and well-executed call to action on a problem which, as the authors correctly identify, has been badly under-appreciated and under-studied by historians. Given the aims of the essay – to raise awareness of the problem and to lay out a potential line of research – I think that it does not need serious revision and will make an important impact in its current state. The nature of their argument is spot-on, and they are correct to call out historians for ignoring the history of marine extraction (though I am already sympathetic on this point!). The comments and suggestions I offer are merely a matter of strengthening the case as it stands, and highlighting strengths and concerns which I am sure the authors have already thought about but which stood out to me as a reader.

 An important contribution of this piece is to bring together and summarize several recent lines of research by different teams of historians and archaeologists in Europe .There is considerable value in just laying out the problem as it is now understood by fisheries historians and archaeologists. The notes are in and of themselves a major contribution to the scholarship, acting as a kind of ersatz bibliography of the latest research on marine environments and extraction. It is particularly striking that the authors range so broadly across time and space in the scholarship they bring together, in a way which is unusual and helpful for those wishing to begin research projects or teaching endeavors on the topic of marine history.

 A few thoughts:

1. It is not always clear what is the relationship between the call to action and specific projects with which the authors are affiliated. Is this meant to be the manifesto of the emerging 4Oceans project? A preliminary to a set of conferences and funded research? Or a more general, global plea for more research?
2. The long section entitled “The driving forces of marine exploitation” may be the best concise overview of the problem of human-marine extraction interactions yet written. I am probably going to reference this excellent summary in my own writing, and would be very interested in using it for teaching. I would suggest the authors consider foregrounding this section at the start of the article – putting the broad overview before the breakdown of project and approach. It is a passionate, synthetic, and detailed explanation which I think would better frame the case this essay tries to make if placed further up.
3. I am very happy that the authors wish to understand the transition to fishing regimes, including in very early human history. It is not always clear, however, how far back they want to go – much of the specific examples are from the past two millennia, and I wished for some clarification if they want to go all the way back to the early Holocene and the emergence of fishing/fisheries.
4. While the authors point to many potential lines of inquiry, research projects, and potential subjects, I do think it would be helpful to specify a few key sites/times/topics which could serve as a starting point. What are, perhaps, 4-5 crucial research sites and topics we need to know more about, and which could serve as an initial testing ground for the methods they are proposing? Coastal California, southeast Asian islands, coastal West Africa perhaps? I say this not to be pedantic, but to suggest that the call to action might be more manageable if it was a bit more specific (at least in the near-run). And given the expertise of the authors, I am genuinely curious as to what they see as those topics most in need of scholarly attention.
5. There is some scholarship on this problem from the US and Canada which is not cited, and which may be of use to the authors. It is less quantitative, but I think indicative of the newer research on marine extraction. I am here thinking of work by Jakobina Arch, Brian Paine, Bathsheba Demuth, Arianne Urus, Jack Bouchard, Elizabeth Mancke, etc.
6. On a personal note, I would say the early modern European extractive fisheries along the Saharan Coast of Africa (the Rio do Ouro and Arguim) are badly under-studied and would be a great case-study for this kind of research agenda.

The following is not necessarily something the authors have to address in this piece, but reflects more of my general reaction to this essay: I have one overarching concern from which my other comments will follow. This is that the essay, as it currently stands, spends far more time laying out the problem than the solution. Much of the text is devoted to describing the importance of future fisheries research, and the benefits to be derived from this. The actual discussion of evidence, methods, and plans is much more brief. To put it another way, at the moment the essay is thought-provoking, but I am not sure it is *persuasive. *It is a call to arms which gestures as potential futures, but does not always set out a practical roadmap.  

 I have two interrelated comments/challenges for the authors:

1. I am not sure that the authors’ emphasis on a *quantitative *approach to premodern global extractive histories is realizable, nor does their discussion of evidence suggest that it will be easy, given the nature of the documentary and archaeological sources. I suspect they are equally interested in qualitative approaches, which may be our only options in some time/place settings, but that is not always made clear.
2. I am concerned by the presumption that methods and theories developed largely in northern Europe to study premodern northern Atlantic and northern European marine ecosystems can, or should, be applied globally.

Previous work on premodern fishing, which the authors draw heavily upon, has focused largely on northern Europe and the north Atlantic, with a particular emphasis on evidence from the British Isles, northern German, Scandinavia, and coastal France. They correctly point out that there has been a rising interest in research outside of the global north, but the substantive body of scholarship (especially quantitative) comes from the north. The essay points to work on the Fish Event Horizon (the North Sea zone), NorfFsh project (North Atlantic), Arctic Whaling (Spitsbergen, the Canadian Arctic, Terra Nova, Berengia, etc.), and coastal fisheries in the Baltic. The authors and project team are overwhelmingly scholars of northern Europe and the North Atlantic, and based primarily in the British Isles. (With the significant exception of Portuguese scholars in the project, which raises the question of why Iberian evidence/fishing histories aren’t highlighted more in this project?)

 The section on evidence (pp.4-5) is only about three paragraphs long and, while it summarizes previous work and explains the importance of Big Data approaches, it does not actually address the nature of the global evidence for premodern marine extraction. The documentary evidence that would allow for quantitative approaches is often sparse to nonexistent even before 1600, even in Europe. The 16 ^th^-century data for the Newfoundland/Terra Nova cod fisheries, for instance, is very patchy before the 1580s (leading to an over-reliance on a few cities like Rouen, La Rochelle, and Bordeaux). They point to the success of whaling studies, but this only proves the problem I am trying to raise: that research was possible only because of the unusually rich documentary basis in northern European archives, the unusual investment in archaeology at places like Spitsbergen, and because historians have largely focused on the periods when documents become more available after the early 18 ^th^ century.

 I am somewhat uneasy by the authors’ desire to apply the Fish Event Horizon model to the rest of the world, using the Marine Event Horizon model. This smacks of assuming a northern European phenomenon must be a global norm – and a desire to go looking for it everywhere else on the planet. I am not persuaded that the MEH will actually prove to be a global norm, or that we should, in effect, go looking for North Seas everywhere else. I assume that is not their intention, but the essay gives this scant mention. I would like to see more discussion of how the authors intend to explore, refine, and accommodate alternative models and frameworks for understanding changing extractive regimes in a global context.

 To give a concrete example to this, on page 8 (of the pdf) the authors point to the potential to apply their methods to California’s channel islands. I find that a very appealing prospect, but am not sure how it would be realized. How do we overcome the absence of archival sources, and rely solely on archaeology? Has there been sufficient archaeological work, and if not, how do we remedy that? Can we translate this into quantitative data? And if we go looking in California for a MEH, what do we do if we don’t find it? I imagine the authors have answers to all these questions, and will no doubt address them in future work.

 The authors gesture to a desire to expand their research towards the global south. I could not agree more, and I am delighted – thrilled, even – to see this as a core part of their research agenda. To me, nonetheless, this raises a few concerns:

1. The serious shortfall in archaeological work in many parts of the world, resulting from longstanding systemic biases against funding and organizing such efforts. This is obviously not the fault/problem of the authors, but it is a real stumbling block. There has been, for instance, much more archaeological work specifically focusing on marine extraction done around the North Sea basin than around, say, Chesapeake Bay, the Gulf of St. Lawrence, coastal Saharan Africa, the islands of the Indian Ocean, or the southern rim of the South China Sea. It’s not clear if the authors want to fund/organize more global research, or if they mean to draw on what already exists? How do we work with and close this gap?
2. The archival gaps which may exist for such a quantitative study, or the potential for archival materials. The archaeological work on northern Europe shows one potential route around this, but has always been put in dialogue with documentary sources – what do we do where we must rely solely on archaeological work?  
3. The language barriers this poses to a team based primarily in western/northern Europe – how do we engage with scholarship and sources from beyond western archives? Should a new generation of fisheries historians be trained in non-European languages?
4. Is this is a call to get more scholars from the global south involved with maritime/fisheries history? (If so, what would that look like – should there be more funded PhDs available to bring scholars from the global south to universities in Europe, the UK, and US/Canada?) Or is it a call for scholars from the global north to pay more attention to the global south? The latter seems to be the case, but perhaps it is time for the former?

Again, I offer these thoughts not as a critique necessarily but as a set of ideas which the authors might want to mull over as the project moves forward. The piece is overall excellent, and I am happy to see it will soon be formally out in the world and open access.

Is the argument persuasive and supported by appropriate evidence?

Yes

Is the work clearly and cogently presented?

Yes

Is the topic of the essay discussed accurately in the context of the current literature?

Yes

Does the essay contribute to the cultural, historical, social understanding of the field?

Yes

Reviewer Expertise:

I research 15th-16th century Atlantic fisheries, especially Terra Nova/Newfoundland, and work on global premodern marine environments more generally

I confirm that I have read this submission and believe that I have an appropriate level of expertise to confirm that it is of an acceptable scientific standard.
